# The mismatched nucleotides encoded in vaccinia virus flip-and-flop hairpin telomeres serve an essential role in virion maturation

**DOI:** 10.1371/journal.ppat.1010392

**Published:** 2022-03-15

**Authors:** Mira M. Shenouda, Ryan S. Noyce, Stephen Z. Lee, Jun Li Wang, Yi-Chan Lin, Nicole A. Favis, Megan A. Desaulniers, David H. Evans

**Affiliations:** 1 Department of Medical Microbiology & Immunology; 2 Li Ka Shing Institute of Virology, University of Alberta, Edmonton, Alberta, Canada; University of Florida, UNITED STATES

## Abstract

Poxvirus genomes consist of a linear duplex DNA that ends in short inverted and complementary hairpin structures. These elements also encode loops and mismatches that likely serve a role in genome packaging and perhaps replication. We constructed mutant vaccinia viruses (VACV) where the native hairpins were replaced by altered forms and tested effects on replication, assembly, and virulence. Our studies showed that structure, not sequence, likely determines function as one can replace an Orthopoxvirus (VACV) hairpin with one copied from a Leporipoxvirus with no effect on growth. Some loops can be deleted from VACV hairpins with little effect, but VACV bearing too few mismatches grew poorly and we couldn’t recover viruses lacking all mismatches. Further studies were conducted using a mutant bearing only one of six mismatches found in wild-type hairpins (SΔ1Δ3–6). This virus grew to ~20-fold lower titers, but neither DNA synthesis nor telomere resolution was affected. However, the mutant exhibited a particle-to-PFU ratio 10-20-fold higher than wild-type viruses and p4b/4b core protein processing was compromised, indicating an assembly defect. Electron microscopy showed that SΔ1Δ3–6 mutant development was blocked at the immature virus (IV) stage, which phenocopies known effects of I1L mutants. Competitive DNA binding assays showed that recombinant I1 protein had less affinity for the SΔ1Δ3–6 hairpin than the wild-type hairpin. The SΔ1Δ3–6 mutant was also attenuated when administered to SCID-NCR mice by tail scarification. Mice inoculated with viruses bearing wild-type hairpins exhibited a median survival of 30–37 days, while mice infected with SΔ1Δ3–6 virus survived >70 days. Persistent infections favor genetic reversion and genome sequencing detected one example where a small duplication near the hairpin tip likely created a new loop. These observations show that mismatches serve a critical role in genome packaging and provide new insights into how VACV “flip and flop” telomeres are arranged.

## Author summary

Poxviruses employ linear double-stranded DNA genomes that end in incompletely base-paired hairpin termini. These mismatched ends are thought to serve some role in virus assembly, and perhaps replication, but have not been amenable to genetic analysis. In this study we used a synthetic virology approach to alter the sequence and structure of these elements. Our research shows that although the encoded structures are of critical importance for function, the sequences are not because one can swap the ends of viruses from different poxviruses without affecting growth. When one tries to progressively delete the mismatches that are found at these ends (the telomeres) of wild-type genomes, it creates an assembly defect which shows up as an increase in the number of virus particles per infectious unit and an accumulation of incompletely assembled viruses. Electron microscopy showed that the development of mutant viruses is blocked at a stage after DNA is packaged but before the particles fully mature. This investigation supports earlier studies that had identified the telomeres as being sites where virus proteins bind and promote packaging. Viruses bearing these mutant telomeres are also less virulent but can still serve as vaccines to protect mice from a lethal virus challenge.

## Introduction

Poxviruses encapsulate large double-stranded DNA genomes which terminate in incompletely base paired hairpin ends. These mismatched hairpins are found in all of the poxviruses that have been completely sequenced, however their structure, sequence, and length varies. Although the existence of these intriguing structures has been known for nearly 40 years [1], the various roles they might play in virus biology has not yet been fully elucidated. The first of these telomere ends to be sequenced were those encoded by an Orthopoxvirus, vaccinia virus (VACV) [1]. It was subsequently shown that a Leporipoxvirus, Shope (or rabbit) fibroma virus (SFV), employed similar structures but comprising different sequences [2]. These and other studies have shown that poxvirus hairpin ends are formed from the refolding of two imperfectly complementary inverted repeat sequences, which then assume slightly different structures due to the presence of different mismatches. This difference causes the two species to migrate differently on non-denaturing DNA gels, which have been called fast (F) and slow (S) forms or sometimes also “flip” and “flop” [1].

Earlier studies have looked into the involvement of the hairpin ends in the processes of replication and concatemer resolution. Poxvirus DNA replication produces long concatemers of virus genomes linked in a randomly oriented manner [3], and plasmids encoding cloned fragments of the concatemer junctions have been used to show that these junctions can be extruded into cruciform-shaped Holliday structures bearing hairpin ends. This led Morgan and McFadden to propose that poxvirus hairpin ends are formed when such junctions are cut by a virus-encoded resolvase [4]. The subsequent discovery that VACV A22R encodes a Holliday junction resolvase has confirmed the essential correctness of this model [5].

The process of concatemer resolution has been studied extensively using transfection assays [6–11]. These studies have shown that concatemer resolution requires a nearby element termed a concatemer resolution site (CRS). The CRS is a highly conserved sequence and it is found adjacent to the sequences that form the AT-rich incompletely base paired hairpin ends. The CRS element can act as a late promoter and concatemer resolution depends upon late transcription. Resolution reactions require an inverted repeat lying between the two CRS sites in a concatemer junction, however the sequence does not seem to matter, nor is it clear what role the mismatches might serve. It was also discovered that SFV-infected cells can resolve plasmids encoding SFV or VACV concatemer junctions into linear mini-chromosomes. The same effect was seen in VACV-infected cells although in both cases the homologous resolution reaction seemed to be slightly more efficient than heterologous resolution.

Although it is not clear what role the sequences destined to form the hairpin ends play in concatemer resolution, it is known that VACV encodes at least two DNA binding proteins (I1 and I6) that can recognize the extra-helical bases in mature hairpin structures and play an important role in genome packaging [12–15]. Supressing I1 expression or inactivating a temperature-sensitive I6L allele blocks the conversion of immature to mature virions. Binding does not seem to be sequence specific although it does require at least two mismatched bases *in vitro*. It may also play some role in replication. Earlier studies have shown that any plasmid transfected into a poxvirus-infected cell can be replicated to form high molecular DNA, thus DNA synthesis does not seem to require a specific origin of replication [16]. However, mini chromosomes bearing ~200 bp of VACV DNA encompassing the hairpins, mismatches, and the CRS, exhibited a replicative advantage compared with molecules composed of unrelated DNA [13]. This favours an old proposal suggesting that virus replication might initiate and terminate in the telomeres [17] and such a hypothesis is further supported by the discovery of an excess of Okazaki fragments initiating close to the hairpin centre [18].

Despite these significant advances in our understanding of these structures, the fact that they comprise non-canonical duplex DNAs located at the ends of the genome makes it difficult to manipulate the sequence *in situ* using conventional molecular techniques. Past research has thus mainly used transfection assays. It is not known whether such an approach accurately reproduces the properties of these virus-encoded structures, nor how these elements affect virus viability and the fitness. We have recently described a fully synthetic method for assembling recombinant Orthopoxviruses and it has opened up new opportunities for investigating the properties of poxvirus genomes. Thus, in this study we examined the effects of changing the sequence and structure of the hairpin ends on the viability and fitness of VACV. The results suggest that altering the structure, and thus the stability, of the hairpin end can have dramatic effects on the growth and virulence of the virus both *in vitro* and *in vivo* mouse models.

## Results

### Reactivation of a synthetic copy of Acambis 2000 vaccinia virus

**Fig 1:**
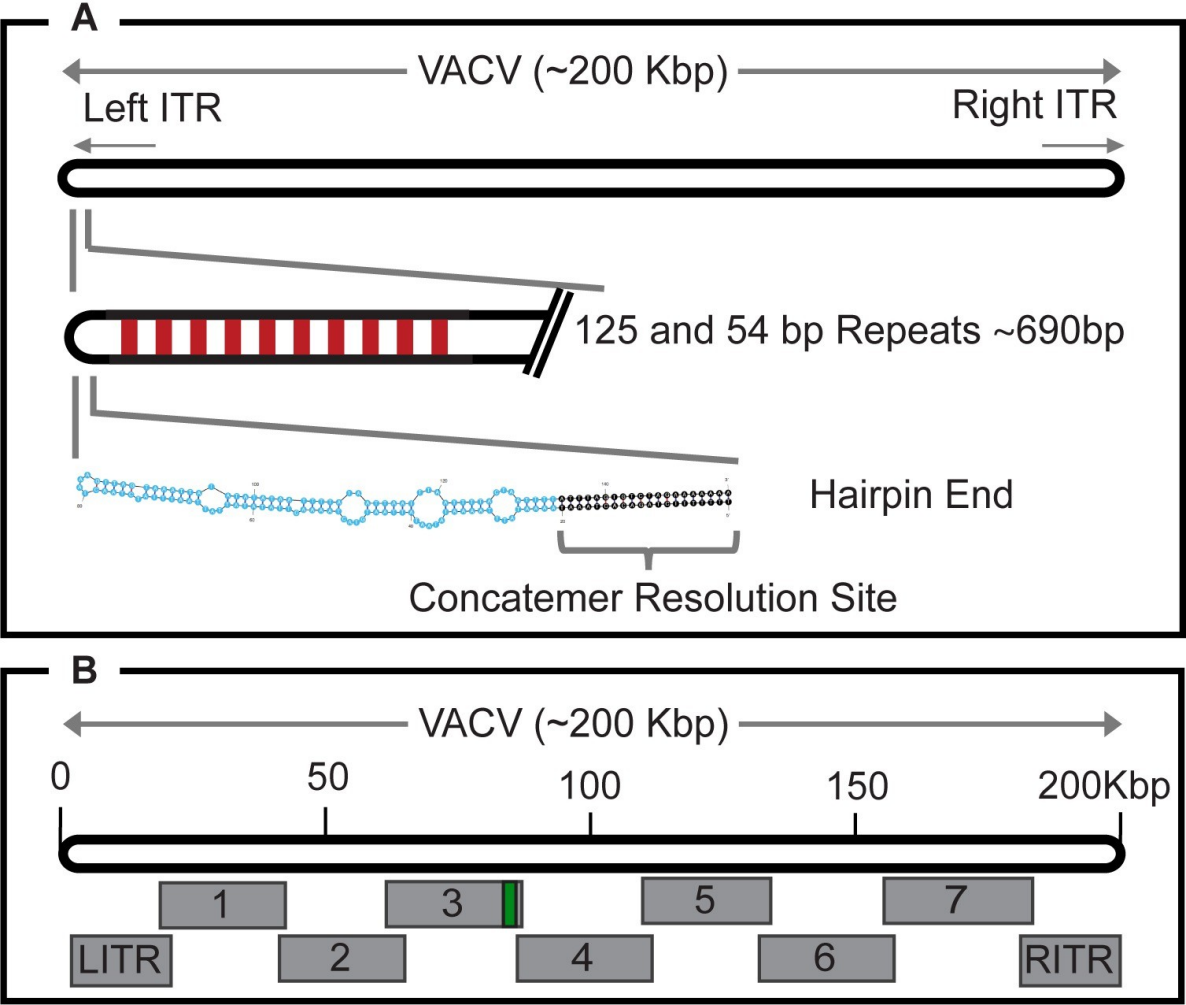
Map illustrating the structure of VACV genomes and the elements discussed in the text. Panel A shows the 125 and 54 bp repeats that lie in-between the hairpin ends of the genome and the first and last open reading frames. “Native” viruses also encode variable numbers of 70 bp repeats that were omitted from the synthetic viruses studied here. The concatemer resolution site (CRS) is a highly conserved element that is required to convert concatemeric replication products into monomer genomes during packaging. All of these elements are embedded within inverted terminal repeats (ITR). Panel B shows the strategy used to assemble the virus. Different forms of hairpin ends were ligated to the left and right ITRs.

We have previously described a method that permits the reconstruction of an infectious Orthopoxvirus using synthetic clones spanning the virus genome [19]. In this study we used the same methods to produce a synthetic copy of VACV strain Acambis 2000 (A2K) and have used this approach to test how the hairpin sequence and extra-helical loops affect virus growth and virulence. A2K was cloned from the once widely used smallpox vaccine, Dryvax, and we elected to use it as a template because it was envisioned that synthetic derivatives of similar viruses might find future utility as viral vectors. We began by sequencing a cloned specimen of A2K virus to confirm the accuracy of the reported sequence (AY313847) and to determine the missing sequences encompassing the 125, 54, and 70 bp repeats [20,21] and the CRS and hairpin ends. The resulting sequence assembly for our “copy of A2K” (cA2K: GB MN974380) spanned about 200 kbp (the number is approximate as the number of 70 bp repeats isn’t certain) and ends in mismatched hairpin structures (Fig 1A). The sequence was divided into nine segments each overlapping by 1 kbp (Fig 1B) and fragment #3 was modified to incorporate a selection marker in the J2R locus (yellow fluorescent protein fused to guanosine phosphoribosyl transferase (YFP-gpt)) [22]. In designing the left- and right-inverted terminal repeat segments we retained the 125 and 54 bp repeats that lie between the telomeres and the first open reading frames but omitted all of the 70 bp repeats. We also substituted the more extensively characterized VACV strain WR hairpins (Table 1) for the cA2K hairpins, even though they differ slightly in sequence. After ligating the WR hairpins to the left- and right-inverted terminal repeat segments, these DNAs and SFV-catalyzed reactivation reactions were used to assemble a synthetic copy of cA2K (sVAC-wt, GenBank: MW960418).

**Table 1:** Oligonucleotides used for hairpin assembly.

Name	Oligonucleotide sequence (5’-3’)^†^
S	pACATTTTTTTCTAGACACTAAATAAAATATTTAAAATATAATATTAATGTACTAAAACTTATATATTATTAATTTATCTAAC**TAAA**GTTAGTAAATTATATATATAATTTTATAATTAATTTAATTTTACTAATTTTATTTAGTGTCTAGAAAAAAA
SFV	pACATTTTTTTCTAGGGTTATAAATTACTTACATAATGTAATTGATAAAAATTAATAAATGATTATATTTATCC**TTAA**GGATAAATTAACATTTCTATTTTTACATTACATTATGTAAGTAATTTATAACCCTAGAAAAAAA
SΔ6	pACATTTTTTTCTAGACACTAAATAAAATTAAAATATAATATTAATGTACTAAAACTTATATATTATTAATTTATCTAAC**TAAA**GTTAGTAAATTATATATATAATTTTATAATTAATTTAATTTTAATTTTATTTAGTGTCTAGAAAAAAA
SΔ5–6	pACATTTTTTTCTAGACACTAAATAAAATTAAAATATTAATGTACTAAAACTTATATATTATTAATTTATCTAAC**TAAA**GTTAGTAAATTATATATATAATTTTATAATTAATATTTTAATTTTATTTAGTGTCTAGAAAAAAA
SΔ1–3	pACATTTTTTTCTAGACACTAAATAAAATATTTAAAATATAATATTAATGTACTAAAATTATATATTAATTTACTAAC**TAAA**GTTAGTAAATTAATATATAATTTTATAATTAATTTAATTTTACTAATTTTATTTAGTGTCTAGAAAAAAA
SΔ3–6	pACATTTTTTTCTAGACACTAAATAAAATTAAAATATTAATTAAAATTATATATTATTAATTTATCTAAC**TAAA**GTTAGTAAATTATATATATAATTTTAATTAATATTTTAATTTTATTTAGTGTCTAGAAAAAAA
SΔ1–5	pACATTTTTTTCTAGACACTAAATAAAATATTTAAAATATTAATTAAAATTATATATTAATTTACTAAC**TAAA**GTTAGTAAATTAATATATAATTTTAATTAATATTTTACTAATTTTATTTAGTGTCTAGAAAAAAA
SΔ2–6	pACATTTTTTTCTAGACACTAAATAAAATTAAAATATTAATTAAAATTATATATTAATTTATCTAAC**TAAA**GTTAGTAAATTAATATATAATTTTAATTAATATTTTAATTTTATTTAGTGTCTAGAAAAAAA
SΔ1Δ3–6	pACATTTTTTTCTAGACACTAAATAAAATTAAAATATTAATTAAAATTATATATTATTAATTTACTAAC**TAAA**GTTAGTAAATTATATATATAATTTTAATTAATATTTTAATTTTATTTAGTGTCTAGAAAAAAA
SΔ1–6	pACATTTTTTTCTAGACACTAAATAAAATATAAAATTAAATTAATTATAAAATTATATATATAATTTACTAAC**TAAA**GTTAGTAAATTATATATATAATTTTATAATTAATTTAATTTTATATTTTATTTAGTGTCTAGAAAAAAA

^†^The concatemer resolution sequence is formed by the underlined nucleotides. The four nucleotides that likely form the terminal hairpin loop are shown in bold font.

### Either an F or S hairpin can support vaccinia virus reactivation

The protocol we used previously [19] involved synthesizing both the F and S hairpins and attaching each separately to the ends of the left- and right-inverted terminal repeat fragments (ITRs), respectively. This was done out of an abundance of caution as the relationships between the ends, the ITRs, and the genome are not known. However, upon some consideration of how these ends likely function, we realized that this was probably unnecessary. This led us to test whether we could reactivate the virus using only one of the two end types ligated onto both the left and right ITR fragments. As suspected, we could reactivate a virus using *either* the WR strain F *or* S forms ligated onto both the left and right ITRs. When we sequenced a virus that was reactivated using only the S hairpins, we detected reads derived from equal numbers of F and S telomeres. These results show that a single viral DNA molecule can be assembled and replicated bearing S- or F-hairpins on both ends of the virus. Subsequent processes, such as randomly distributed symmetrical cleavages near a cruciform (i.e. Holliday) junction, are expected to scramble the distribution of the F- and S-ends among the progeny of that virus. From a practical perspective this observation halved the numbers of hairpin oligonucleotides that needed to be purchased. For the rest of the study, we used only one of the ends to construct any particular recombinant virus. For brevity, the viruses are described below according to the oligonucleotide used in the assembly (e.g., sVAC-wt is often described as bearing the “S” hairpin).

### Reactivation of VACV with Shope fibroma virus (SFV) hairpin ends

**Fig 2:**
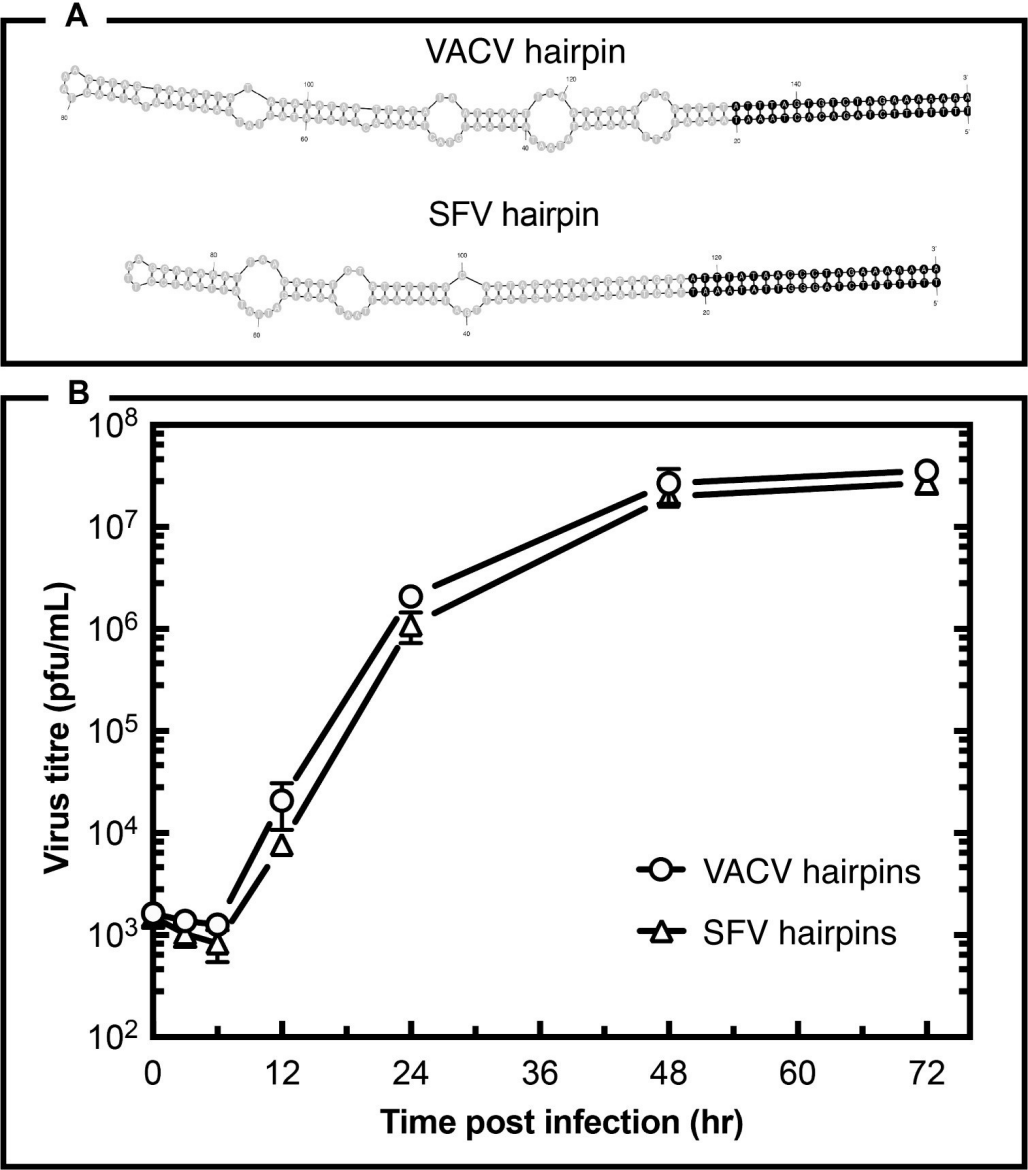
Structure, not sequence, determines the functionality of poxvirus hairpins. Panel A shows the predicted structures of an Orthopoxvirus (VACV) and a Leporipoxvirus (Shope fibroma virus—SFV) hairpin end. The CRS is shown in black font and the sequence, which strongly resembles a poxvirus late promoter, is highly conserved between the two viruses. (Table 1 shows the sequences used to construct the two viruses.) Panel B shows the growth of viruses where the VACV genome was assembled encoding either VACV or SFV hairpins. Each virus titer was determined triplicate on BSC40 cells and error bars are shown where they extend beyond the data points.

Transfection assays have been used to show that VACV-infected cells can process plasmids encoding either VACV or SFV concatemer junctions into linear molecules bearing mismatched and hairpin ends [2]. However, the VACV junctions appeared to be better substrates for VACV catalyzed reactions, suggesting that there is some specificity that favours telomere resolution in the homologous reaction. Closer inspection shows that both the Orthopoxvirus and Leporipoxvirus hairpin ends encode loops and mismatches [2], although the WR hairpin extends further from the CRS than the SFV hairpin and harbours more predicted extra-helical loops within a different sequence (Fig 2A). Between the two flanking CRS elements the two sequences are A+T rich (SFV 84%, VACV 93%) and if gaps are permitted one can align 69% of the residues. However, that’s only marginally more than the 57–65% of bases that can be aligned with random sequences of comparable length and A+T content. These sequence and structural differences provided a substantive test of whether the two elements are interchangeable.

To do this we ligated SFV hairpins onto the left- and right-ITR fragments and tried to assemble an infectious VACV clone as shown in Fig 1B. The added sequences substituted the VACV CRS and WR hairpin termini with the SFV CRS element and its hairpin telomeres. This reaction yielded a number of chimeric VACV clones that were plaque purified free of the SFV helper virus on BSC-40 cells. Genomic sequencing confirmed that the chimeric VACV clone had acquired the SFV CRS and both flip and flop telomeres and, since SFV does not plate on BSC-40 cells (as this is how the helper and reactivated viruses are separated), it provided formal evidence that the telomeres are not a determinant of its host range in BSC-40 cells. We then tested how well this virus (sVAC-SFV) grew compared to VACV bearing WR telomeres (sVAC-wt), by plating the two viruses in parallel at a low multiplicity of infection on BSC-40 cells. There was no significant difference in the rate of growth or final yield of either virus judging by the resulting multi-step growth curve (Fig 2B). This suggests that the SFV hairpin sequence, though different from the VACV sequence, is interchangeable and does not affect the viability and growth of the virus *in vitro*.

### Extra-helical loops are essential

**Fig 3:**
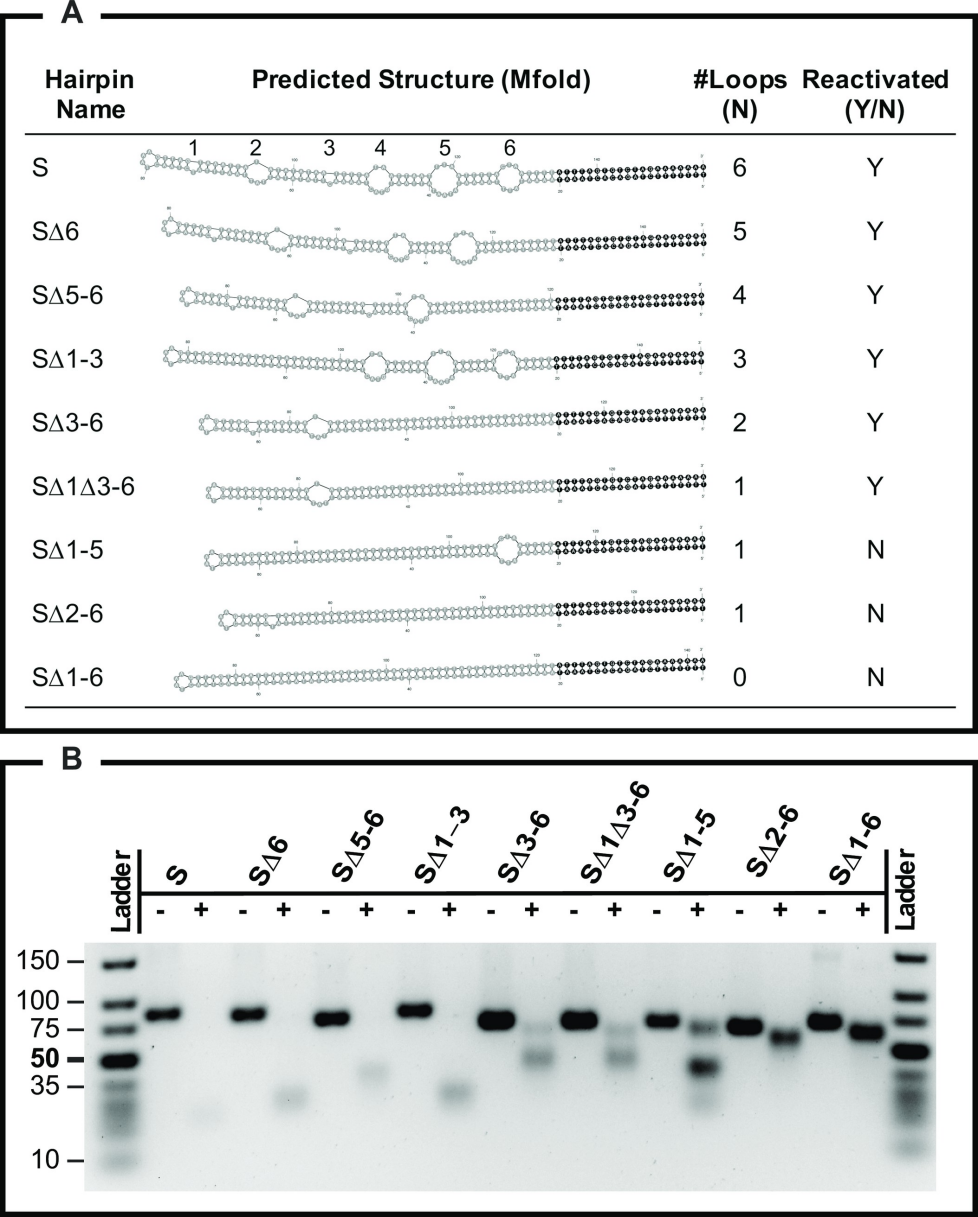
Mutant hairpin elements used in this study. Panel A shows the different hairpin oligonucleotides that were used to try and reactivate VACV. The parent (S) hairpin comes from VACV strain WR. It took two attempts to rescue the SΔ1Δ3–6 tagged virus and three of the hairpins could not be rescued into infectious viruses despite three attempts at reactivation with each. Panel B shows an agarose gel that illustrates the different sensitivities of these oligonucleotides to mung bean exo/endonuclease. Mung bean nuclease is expected to cut at single-stranded sites in the molecules including at the hairpin ends.

Since extra-helical loops are a common and characteristic feature of poxvirus telomeres, we next examined whether the number of extra-helical loops has any effect on virus recovery. We designed a series of hairpin oligonucleotides that lacked some or all of the loops by deleting the nucleotides that mapped to within these loops in the computationally predicted secondary structures (Fig 3A). To show that deleting the different loops caused a corresponding change in the secondary structure of the hairpins, we digested each of the synthetic oligonucleotides with mung bean endonuclease. Each oligonucleotide produced a characteristic pattern of metastable digestion products consistent with the predicted secondary structures (Fig 3B). We next tested whether we could reactivate VACV using the modified hairpins. We could reactivate the virus using hairpins where up to five of the extra-helical loops had been deleted and subsequently confirmed the incorporation of the altered structures into viruses through genome sequencing. We could not, however, reactivate a virus using a synthetic molecule bearing only a single mismatch adjacent to the hairpin end (“loop” 1 in SΔ2–6, although the T is probably an extrahelical base) nor using a DNA bearing only one loop nears the CRS end (SΔ1–5) (Fig 3A). Nor could we reactivate a virus using an oligonucleotide that lacked all of the loops (SΔ1–6). This perfect hairpin was designed slightly differently from the other molecules in that, instead of deleting loop sequences, we copied the complement of the top strand into the bottom strand at loop positions 1-to-5. This eliminated the loops while still retaining much of the linear sequence as well as the hairpin length.

These and the previous results with an SFV hairpin strongly suggest that telomere activity is not determined by sequence but rather by the structure or thermal instability of the mismatched hairpins. M-fold analysis predicted the non-functional and perfect hairpin (SΔ1–6) would be the most stable of these molecules (ΔG = -61 Kcal/mol, 1M NaCl, 37°) while any of the functional molecules bearing two to six loops exhibited ΔGs ranging from -29 Kcal/mol to -47 Kcal/mol for the S and SΔ3–6 molecules, respectively. We struggled to assemble viruses using any of the three molecules encoding a single loop, the one that proved functional after 2 attempts (SΔ1Δ3–6) is predicted to be similar in stability to the other two molecules (-51 versus -51 and -52 Kcal/mol). These three molecules (SΔ1–5, SΔ2–6, and SΔ1Δ3–6,) presumably lie near some critical structure/stability boundary.

### The number of extra-helical loops affects viral fitness

**Fig 4:**
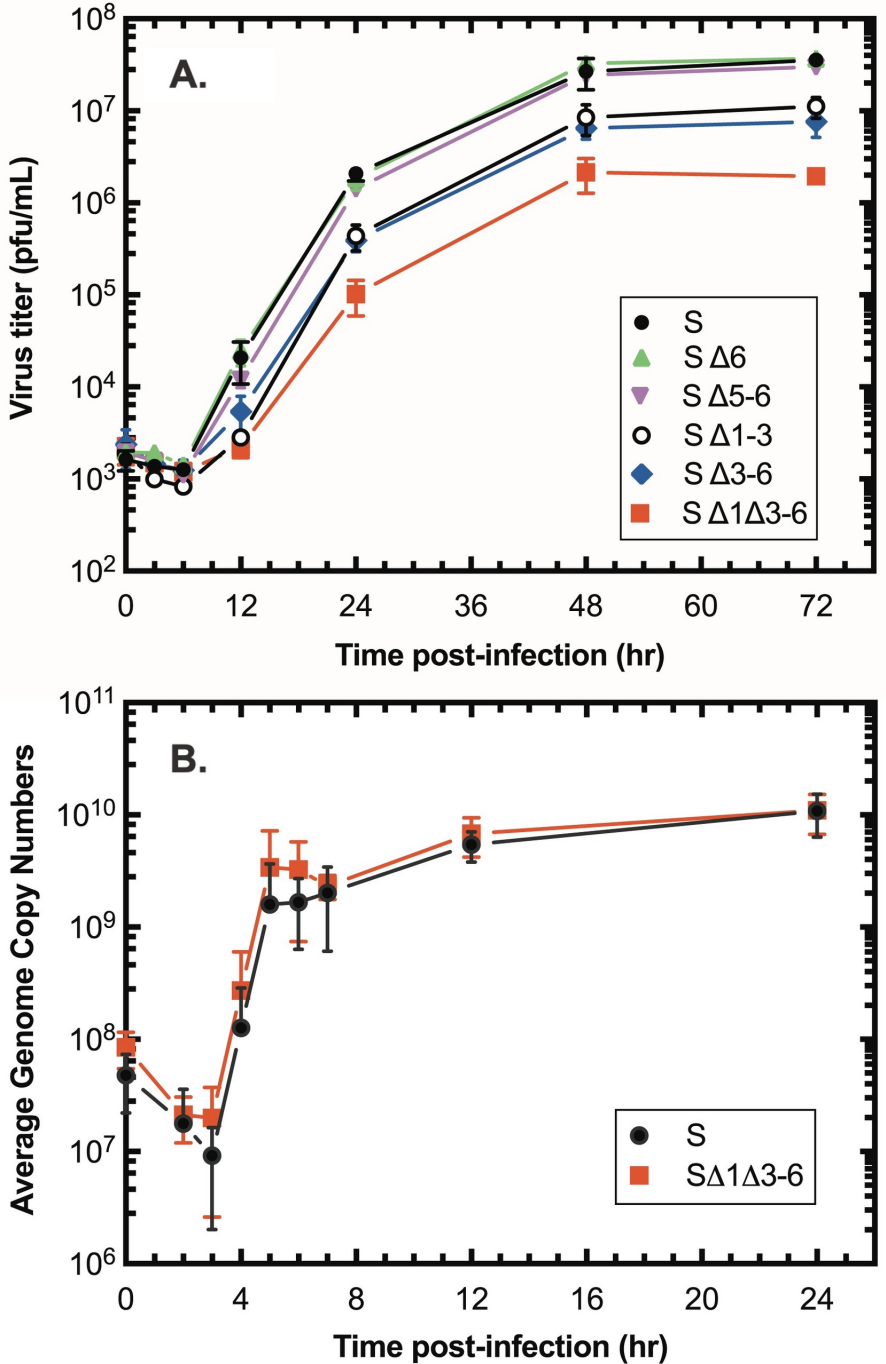
Growth and replication of VACV strains bearing mutant telomeres. Panel A shows multi-step growth curves for each of the indicated VACV strains. Deleting a few of the mismatched loops had little effect on virus growth, but a mutant virus encoding only one of the original loops (SΔ1Δ3–6) showed a significant growth defect. Panel B shows a one-step growth curve comparing DNA replication in cells infected with viruses bearing the wild-type (S) or mutant (SΔ1Δ3–6) telomeres. The number of genome copies was determined using whole cell extracts, qPCR, and E9L gene probes. No obvious defect in DNA synthesis was detected in cells infected with the mutant virus.

We next tested how well these viruses grew in BSC-40 cells infected with virus at a low multiplicity of infection of 0.01. All encoded a ΔJ2R::YFP-gpt insertion mutation in addition to the variant hairpin ends. As the number of the extra-helical loops decreased, we noticed a progressive decrease in the virus yield in these multi-step growth curves, relative to a virus assembled with the wild-type S hairpin (Fig 4A). Loops 5 and 6 appeared to be dispensable in this growth assay, while deleting either the first three (SΔ1–3) or last 3 (SΔ3–6) loops produced some reduction in virus yield. The growth defect was most apparent with viruses encoding only loop 2 (SΔ1Δ3–6), which produced virus titers ~20-fold lower than the control virus. We used this virus (sVAC-Δ1Δ3–6) to further explore what aspect of virus biology was compromised by the mutant telomeres.

One possible explanation for this phenotype is that the mutant hairpins may create a defect in genome replication. To test this hypothesis, we infected cells with either the wild type (S) or mutant (SΔ1Δ3–6) virus at a multiplicity of infection of 3 and harvested the DNA from the infected cells at different times in the infectious cycle. We then used qPCR and E9L gene primers to measure the numbers of VACV genome copies. These data showed that the growth defect cannot be explained by there being a significant difference in the amount of virus genome replication. The cells were infected with nearly equal quantities of virus DNA at time zero (~2-fold less of the wild type) and then the timing, rates, and yields of mutant versus wild-type virus DNA over the subsequent course of infection were essentially identical (Fig 4B).

**Fig 5:**
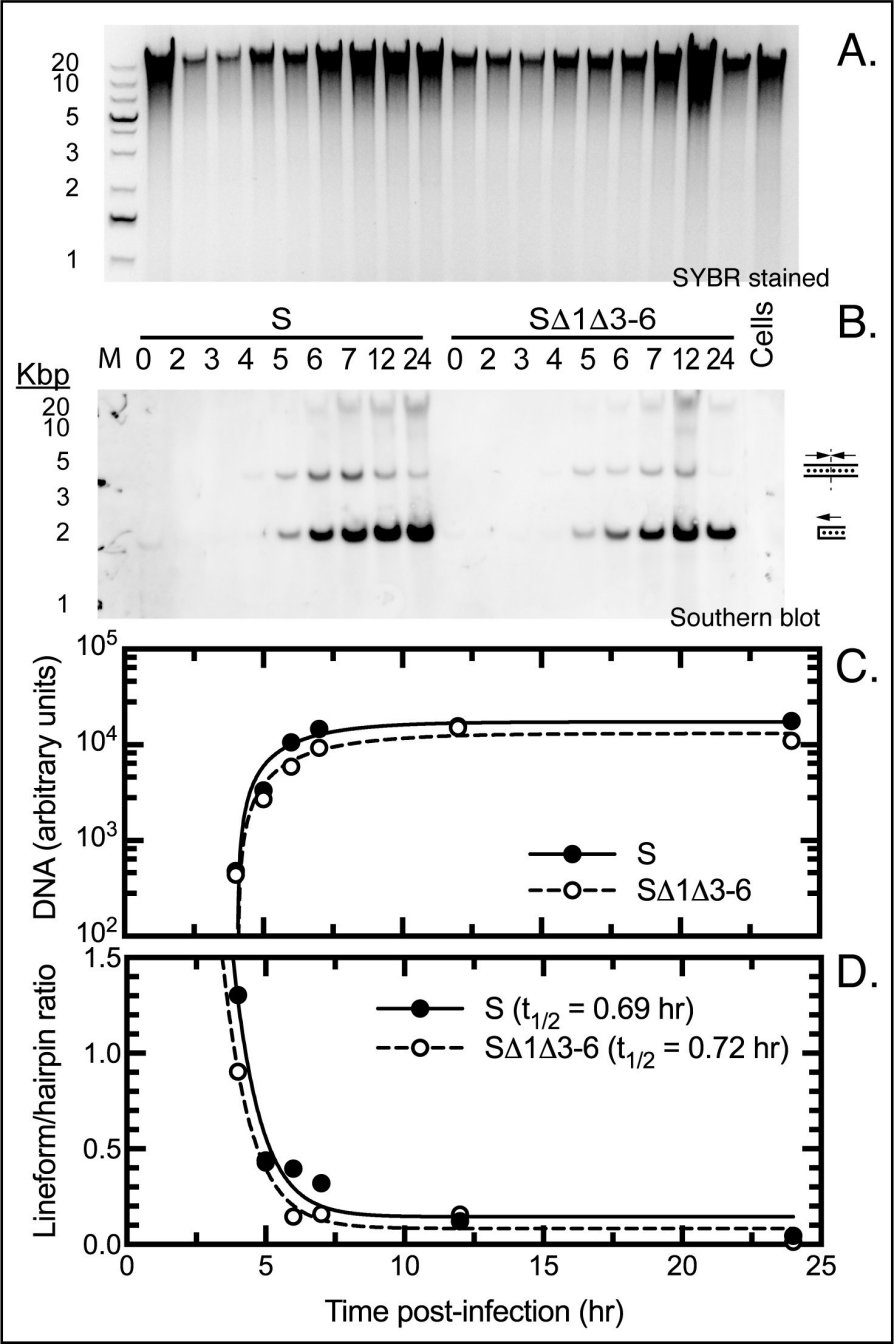
Southern blot showing telomere resolution in VACV-infected cells. The assay detects the conversion of a 4.5 kb concatemeric (line form) replication intermediate into a 2.25 kb hairpin-ended DNA. DNA was extracted from infected cells at the indicated times, digested with *Alw*44I, fractionated by agarose gel electrophoresis (panel A), and Southern blotted using a biotin-labelled VACV telomere probe and chemiluminescent detection kit (panel B). A luminance imager was used to determine the signal intensities which were then used to calculate the amounts of telomeric virus DNA (panel C) and the ratio of lineform-to-hairpin DNA (panel D). Panel D shows two non-linear curves fitted using unconstrained one-phase decay models (R^2^≥0.95). There was no significant difference between the two calculated half-lives (panel D, inset) and no apparent defect in concatemer resolution in cells infected with the SΔ1Δ3–6 virus.

We next tested whether those mutations can alter genome resolution. The same DNA samples that were prepared for the qPCR studies (Fig 4B) were digested with *Alw*44I, the products fractionated through a 0.8% agarose gel (Fig 5A), and Southern blotted using a probe that hybridized to the terminal fragments (Fig 5B). The digest is expected to produce a 4.5 kbp fragment of DNA derived from the unresolved concatemer junctions, or a 2.25 kbp fragment encoding the mature hairpin ends. Based on the data from this and two replicate experiments, there did not seem to be any substantive difference in the efficiency or timing of concatemer resolution (Fig 5D), which also closely tracked the kinetics of DNA replication (Fig 5C). The line-form was the most abundant species detected at the earliest timepoints in these studies, and it was quantitatively converted to the hairpin form with indistinguishable kinetics in cells infected with mutant and wild-type viruses.

### The sVAC-Δ1Δ3–6 virus exhibits an assembly defect

**Fig 6:**
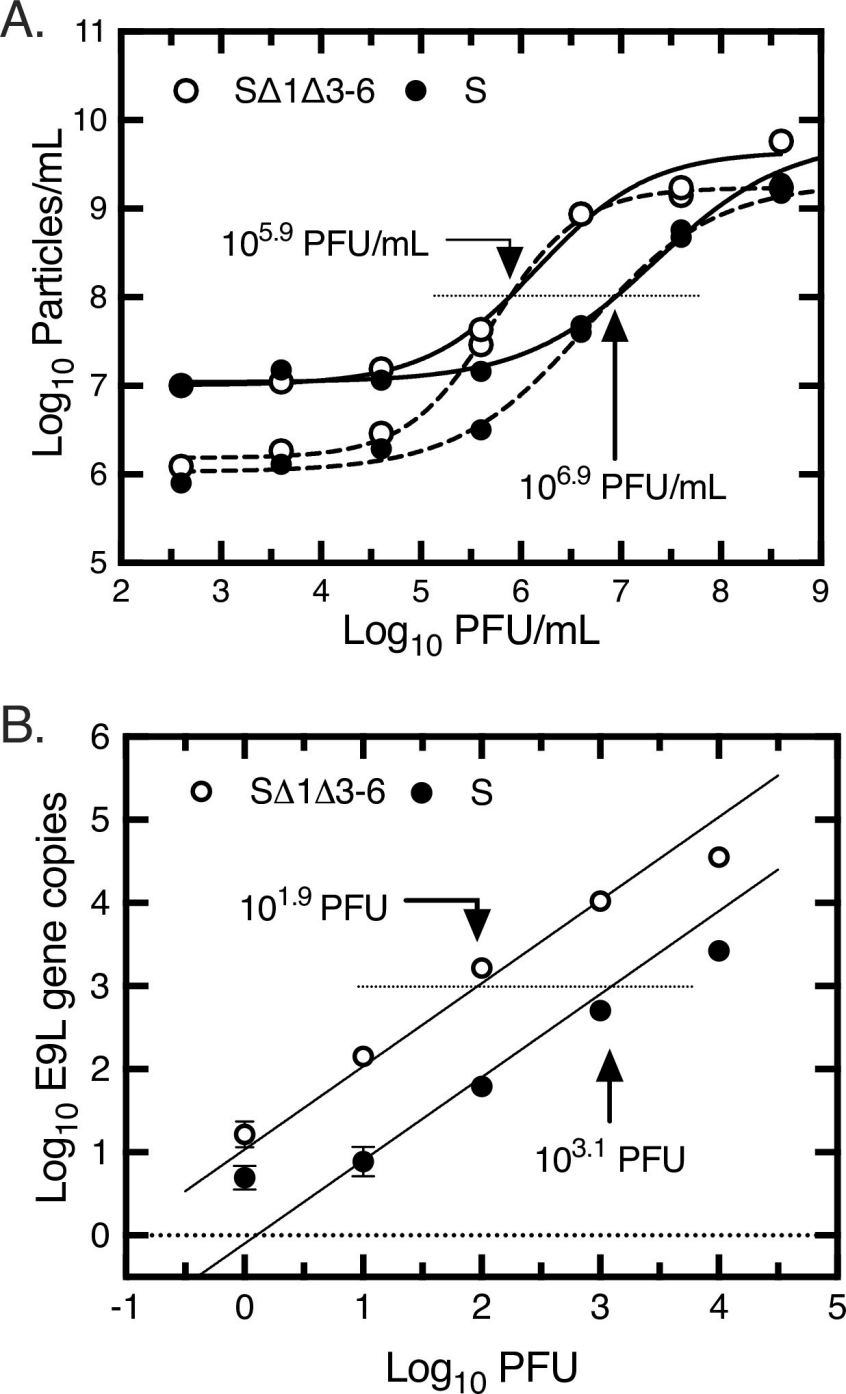
Measurement of particle-to-PFU ratios. Panel A. Flow virometry. Stocks of the sVAC-wt and sVAC-Δ1Δ3–6 viruses were grown and purified in parallel using centrifugation through a sucrose cushion. Ten-fold serial dilutions of each virus were prepared, fixed, and analyzed using a Cytoflex cytometer. Some aliquots were separately stained with SYBR dye. VACV particles in the >180 nm size range were counted using either SYBR dye fluorescence plus light scattering (dashed lines), or using light scattering alone (solid lines). For each data set a non-linear best fit to a sigmoidal curve was calculated using Graph Pad Prism. The figure illustrates the virus titer that corresponds to a particle count of 10^8^ particles/mL (dotted line). Panel B. DNA content. Ten-fold serial dilutions of sVAC^YFP-A5^-wt and sVAC^YFP-A5^-Δ1Δ3–6 viruses were prepared and a 5 μL aliquot added directly to each qPCR assay mix. The number of copies of E9L was determined from known quantities of template amplified in parallel. For each data set a best fit line to the log-transformed data was calculated assuming both slopes equaled one. The figure illustrates the number of PFU associated with 10^3^ gene copies (dotted line).

Previous studies have shown that mutating or blocking the expression of the VACV I1 and I6 mismatch binding proteins can inhibit virus maturation [14,15]. More specifically one observes a failure to properly assemble immature virus (IV) particles and convert them into mature virus (MV). For example, preparations of a tsI6-12 virus collected at the non-permissive temperature comprised an abundance of defective and empty (i.e. DNA free) IV particles [14]. This led us to speculate that the SΔ1Δ3–6 virus might share these same assembly defects, although the phenotype would perhaps not be as fully penetrant as null or temperature-sensitive gene mutations. To test this hypothesis, we co-purified stocks of the sVAC-wt and sVAC-SΔ1Δ3–6 viruses and measured the particle-to-PFU ratios using flow virometry and both SYBR-stained and unstained particles (Fig 6A). Poxviruses are well suited for such measurements although these instruments have a limited counting zone (centered on ~10^8^ particles/mL) wherein errors caused by particulate noise and undercounting are minimized. Within this zone, 10^8^ particles/mL were counted in samples containing 10^5.9^ and 10^6.9^ PFU/mL for the sVAC-Δ1Δ3–6 and the sVAC-wt viruses, respectively. This is about 130 and 13 particles/PFU, respectively; a 10-fold difference. As a further check on the method, we used recombination to assemble fluorescent viruses encoding the YFP reporter fused to the A5 core protein [23] in a J2R^+^ background (Table 2) as this permits direct detection of the particles. We measured the same ~10-fold differences in particles/PFU.

**Table 2:** Vaccinia viruses used in this study.

Virus name	Genbank	Comment	Marker
cA2K	MN974380	Cloned from A2K stock (Dr. M. Buller)	none
Western Reserve (WR)	AY243312.1	From American Type Culture Collection	none
sVAC-wt	MW960418	VACV WR hairpins, CRS, and linker	YFP-gpt
sVAC-SFV	MW960419	Shope fibroma virus hairpins, CRS, and linker	YFP-gpt
sVAC-Δ6	MW960420	See Fig 3 (5 loops)	YFP-gpt
sVAC-Δ5–6	MW960421	See Fig 3 (4 loops)	YFP-gpt
sVAC-Δ1–3	MW960422	See Fig 3 (3 loops)	YFP-gpt
sVAC-Δ3–6	MW960423	See Fig 3 (2 loops)	YFP-gpt
sVAC-Δ1Δ3–6	MW960424	See Fig 3 (1 loop)	YFP-gpt
sVAC^NM^-wt	-	Repair of J2R locus in sVAC-wt	none
sVAC^NM^-Δ1Δ3–6	-	Repair of J2R locus in sVAC-Δ1Δ3–6	none
sVAC^A5-YFP^-wt	-	From sVAC^NM^-wt. A5L replaced with a gene encoding a YFP-A5 fusion protein [23]	YFP-A5
sVAC^A5-YFP^-Δ1Δ3–6	-	From sVAC^NM^-Δ1Δ3–6. A5L replaced with a gene encoding a YFP-A5 fusion protein [23]	YFP-A5

Finally, we also used quantitative PCR to calculate the number of E9L gene copies per PFU (Fig 6B) using these additional stocks of sVAC^YFP-A5^-wt and sVAC^YFP-A5^-Δ1Δ3–6 strains. These experiments used the same PCR primers, quantitative methods, and E9L gene calibration standards described in Fig 4. This independent approach showed that 10^3^ copies of the E9L gene corresponded to 10^1.9^ and 10^3.1^ PFU of sVAC-Δ1Δ3–6 and sVAC-wt viruses, respectively (Fig 6B). This is about 13 and 0.8 particles/PFU or approximately 17-fold difference. Collectively these two approaches and additional replicates, using different virus preparations, showed that although the SΔ1Δ3–6 virus particles contain DNA, the mutant virus is proportionately less infectious than the wildtype by a factor of approximately 10-20-fold.

### Mutant VACV exhibit a defect in intracellular mature virus (MV) formation

**Fig 7:**
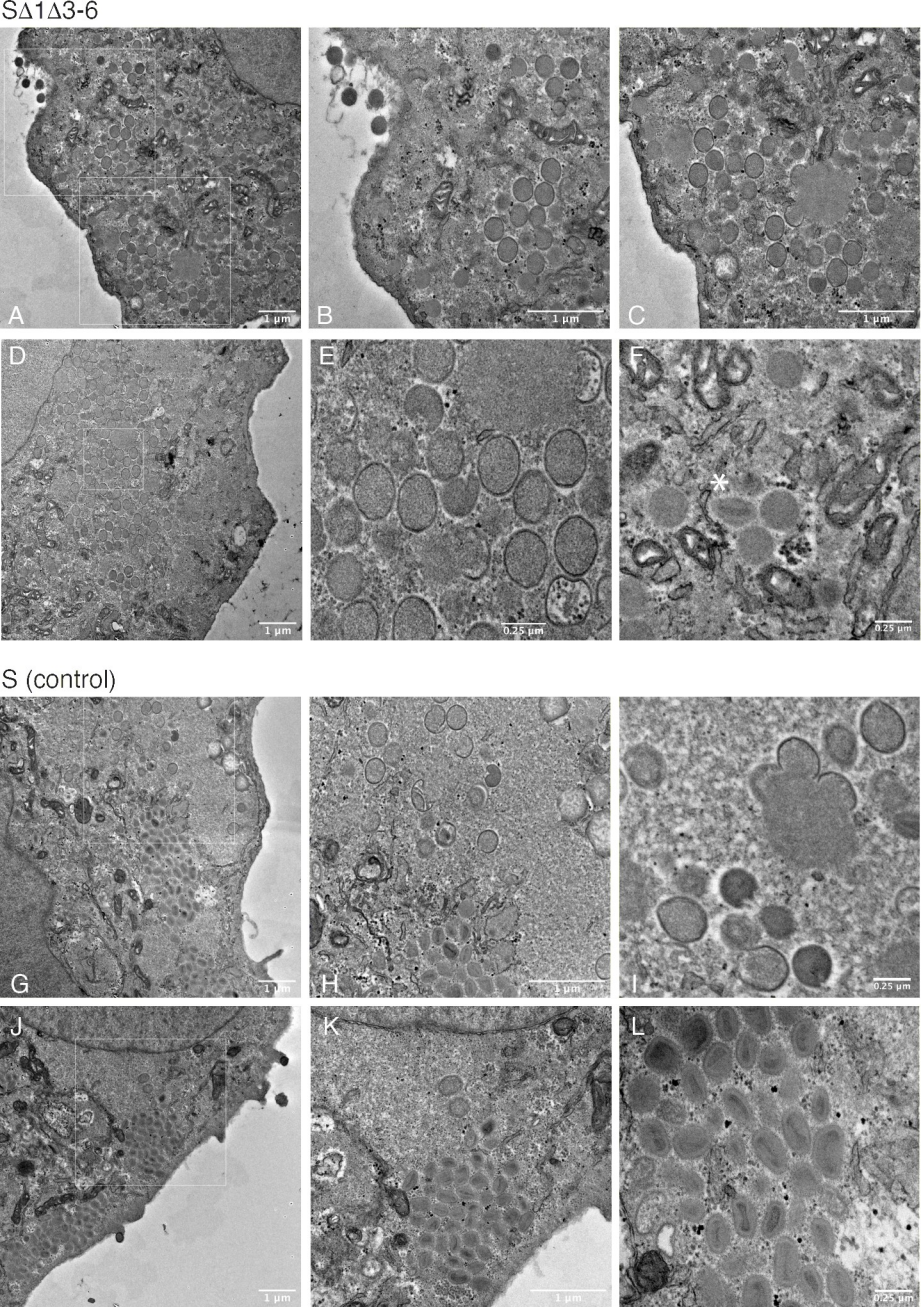
Transmission electron micrographs. BSC-40 cells were infected with the indicated viruses at MOI = 3 for 24 hr and then fixed, processed, and imaged. Panel A. Low-magnification view of a cell infected with the sVAC^A5-YFP^-Δ1Δ3–6 virus. White boxes show regions enlarged in panels B and C. Most of the particles appear to be immature virus (IV) although assembly intermediates are seen adjacent to regions of viroplasm (C). Panel D shows another cell again filled with IV, the area boxed in white is shown enlarged in panel E. Panel F shows a rare example of a particle resembling a mature virus MV (asterisk). Panel G. Low magnification view of a cell infected with the sVAC^A5-YFP^-wt control virus. The area boxed in white is shown enlarged in panel H, where a mix of IV and MV particles can be seen near the viroplasm. Panel I shows another site of virus assembly, the IV particles seen in this and other fields closely resemble those seen in sVAC^A5-YFP^-Δ1Δ3–6 infected cells. Panel J shows another cell filled with predominantly MV forms, this is more clearly seen in the enlargement in panel K. Panel L shows a field of MV imaged at a magnification like that in panel F.

These accumulated data suggested that interfering with the structure of the VACV telomeres creates an alteration that reduces the infectivity of poxvirions. To examine this question in more detail, we used transmission electron microscopy and virus particles harvested at 24 hr post-infection when DNA replication has ceased (Fig 4). These experiments showed that cells infected with the sVAC^YFP-A5^-Δ1Δ3–6 virus contained an abundance of particles resembling the immature virus (IV) form (Fig 7). At 24 hr post-infection these comprised nearly all of the virions seen in SΔ1Δ3-6-infected cells (Fig 7, panels A-F) and were indistinguishable in size and appearance from the small proportion of spherical IV forms still detected at 24 hr post-infection in cells infected with the sVAC^YFP-A5^-wt virus (326±19 x 256±13 nm *versus* 331±23 x 259±7 nm for mutant and control respectively). The mature virus (MV) form was the most abundant type seen in cells infected with the control virus (Fig 7, panels G-L), while only a few rare examples of the MV form were detected in the SΔ1Δ3-6-infected cells imaged in this study (Fig 7, panel F). Some IV-like particles were also seen accumulating on the exterior of cells infected with SΔ1Δ3–6 virus (Fig 7, panel B), but these can also be seen in cells infected with the control.

**Fig 8:**
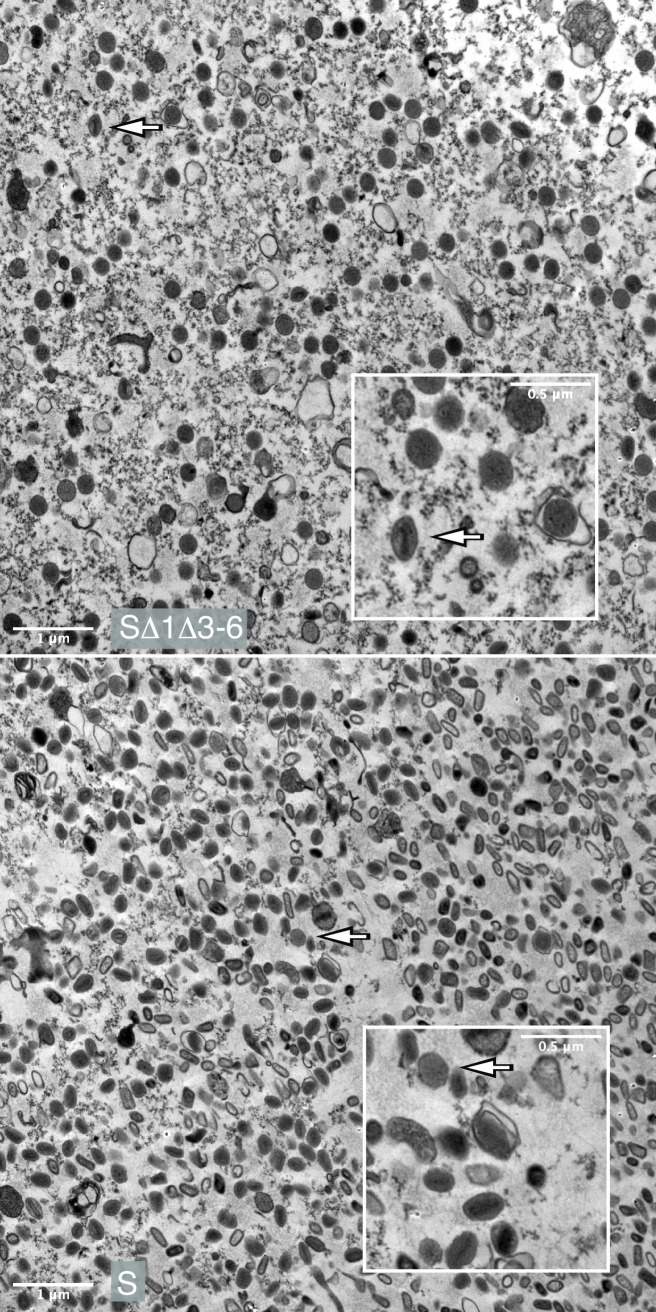
Transmission electron micrographs of virus particles. Concentrated samples of the sVAC^A5-YFP^-Δ1Δ3–6 and the sVAC^A5-YFP^-wt control virus were fixed and embedded in agarose and then processed and imaged as described in Fig 7. Total particle counts in each field (~190 sVAC^A5-YFP^-Δ1Δ3–6 and ~420 sVAC^A5-YFP^-wt) were estimated using Fiji. The sVAC^A5-YFP^-Δ1Δ3–6 virus specimen contained small numbers of oval particles exhibiting an appearance characteristic MV (arrowed) and shown enlarged inset. The sVAC^A5-YFP^-wt control also contains a few spherical particles resembling IV (arrowed and boxed), although given the light contrast it’s possible to confuse these with some orientations of MV.

In order to relate these data to the calculated particle-to-PFU ratios, we used electron microscopy to image particles found in viruses that had been harvested at 3-days post-infection and purified through as sucrose cushion. These micrographs were obtained by embedding the virus in agar, then fixing and sectioning the samples. The images were scanned and counted looking for spherical and elliptical particles in the 0.01–0.07 μm^2^ size range. These experiments showed that the distribution of virus forms in the stocks (Fig 8) reflected what was seen in infected cells (Fig 7). A rough estimate suggested that the sVAC^YFP-A5^-Δ1Δ3–6 stock contained ~97% IV (spherical forms with featureless cores) and 2–3% MV (asymmetric forms usually exhibiting core structures). In contrast the sVAC^YFP-A5^-wt stock comprised 96–98% MV. These estimates suffer from some imprecision as image interpretation is a subjective task, however the 2–3% residual MV that can still be detected in the S1Δ3–6 preparations would account for the ~20-fold reduction in titer (Fig 4) and 10-20-fold reduction in infectivity (Fig 6).

### Core protein processing is suppressed in sVAC-Δ1Δ3–6 viruses

**Fig 9:**
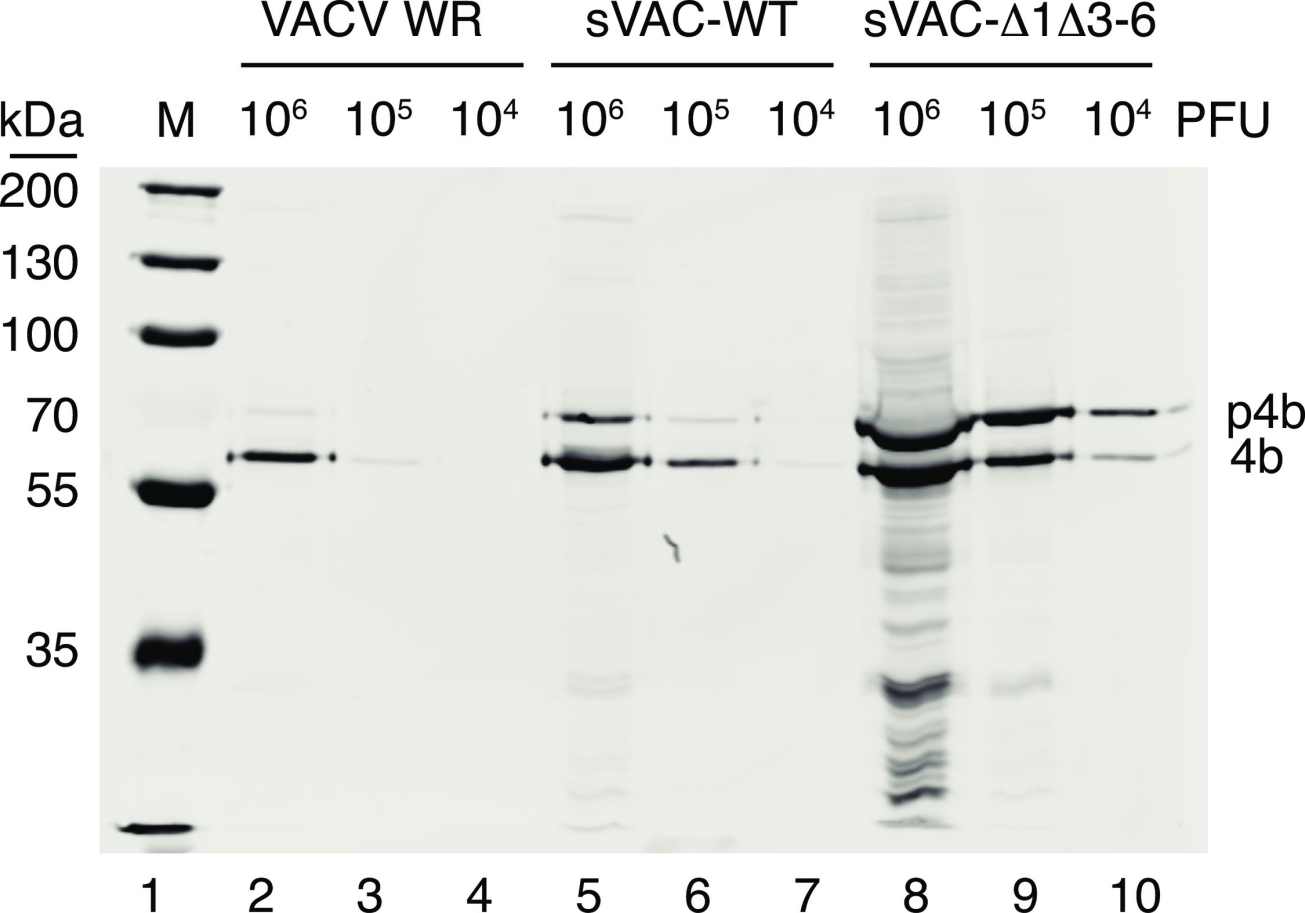
Reduced proteolytic processing of the A3 (p4b/4b) protein. The indicated quantities of each purified virus stock were denatured in loading buffer and size fractionated using SDS-PAGE. The separated proteins were western blotted to detect the p4b precursor and the cleaved 4b product. The amounts of virus loaded in each lane were calculated from the virus titers and because of the 10-20-fold excess of particles in the sVAC^YFP-A5^-Δ1Δ3–6 virus stock (Fig 6), we see a corresponding excess of p4b/4b protein in lanes 8–10 compared to the controls in lanes 2–4 and 5–7. We compared the band intensities in lanes bearing comparable amounts of p4b/4b protein (lanes 2, 6, and 10). This analysis showed that >90% of the p4b/4b protein is cleaved to the 4b form in preparations of the two viruses bearing wildtype hairpins, but only ~30% of the protein is processed to the 4b form in the sVAC^YFP-A5^-Δ1Δ3–6 virus stock.

The conversion of IV to MV is associated with the proteolytic processing of five core virus proteins [24]. A3L encodes a 73 kDa precursor protein that’s one of these proteins, called p4b, that is processed into a 60 kDa protein called 4b. We used a polyclonal antibody and a western blot to detect the relative proportion of the two forms in stocks of different viruses (Fig 9). These experiments showed that >90% of the p4b/4b protein was converted into the 4b form in stocks of VACV strain WR and the sVAC^YFP-A5^-wt control strain (lanes 2 and 6). In contrast, we detected only 30% conversion of the p4b/4b protein to 4b in viruses from a sVAC^YFP-A5^-Δ1Δ3–6 stock (lanes 8–10). The block in converting IV to MV forms in viruses lacking the proper telomeric mismatches is accompanied by a coincident defect in processing of the p4b core protein.

### Mutant hairpin DNAs bind poorly to the I1 telomere-binding protein

Two VACV proteins are known to bind to hairpin DNAs bearing mismatched sequences, I1 and I6 [12]. Disrupting I6 function produces a DNA encapsidation defect, whereas suppressing I1 expression causes an accumulation of IV. The phenotype observed in sVAC-Δ1Δ3-6-infected cells consequently phenocopies an I1L mutation. To test whether the growth (or non-recovery) of these viruses correlates with protein binding we constructed bacterial expression vectors encoding codon-optimized and his_6_-tagged versions of I1L and I6L. I6 formed intractable inclusion bodies, and so for the purpose of this study we focused our attention on the more relevant I1 protein which was easily purified in a soluble form. A gel-shift assay was used to study protein binding, as previously described [12], except that the hairpin oligonucleotides (Fig 3) were 3’-end-labeled with Cy5-dUTP and imaged fluorescently. All the binding assays were performed in the presence of an excess of competing poly·d(IC), also as previously described.

**Fig 10:**
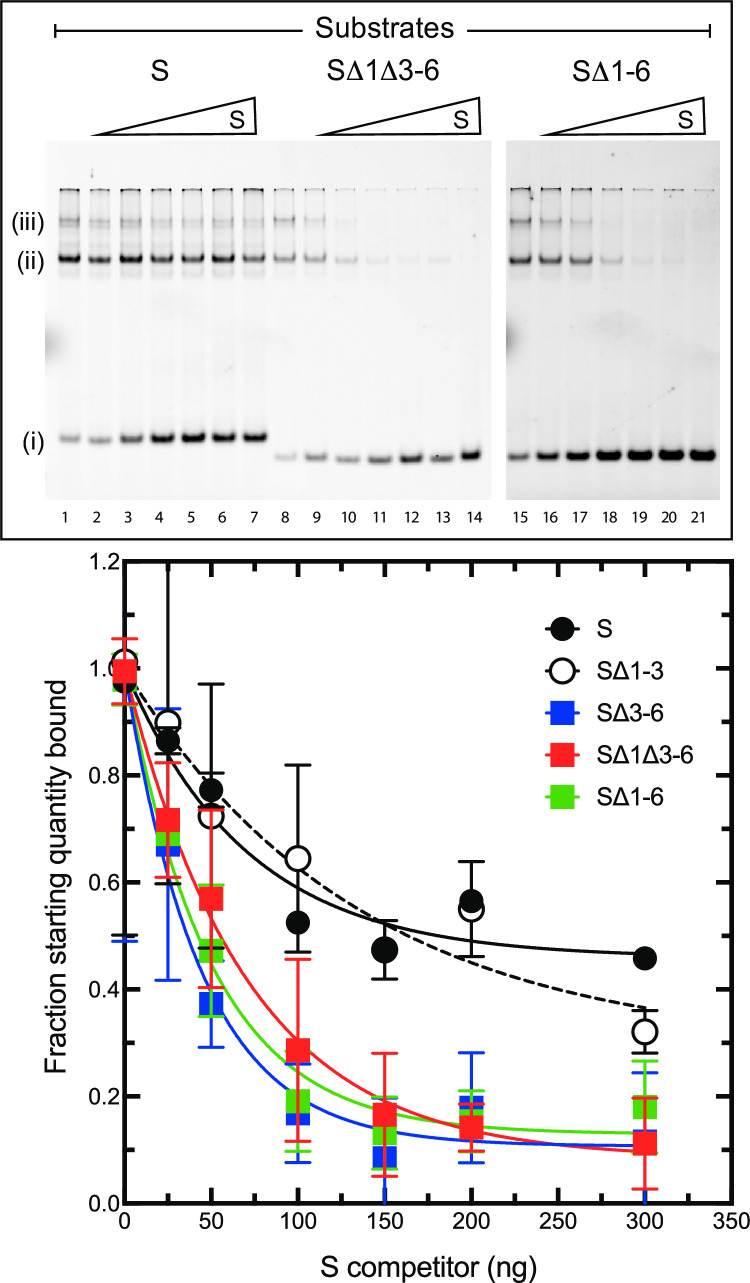
VACV I1 protein binding to selected hairpin oligonucleotides. Each binding reaction contained ~100 ng of recombinant I1 protein, 100 ng of fluorescently-labelled hairpin oligonucleotide (as indicated), 700 ng of a non-specific poly·d(IC) competitor, and 0–300 ng of unlabeled S hairpin. Once assembled, the DNA-protein complexes were electrophoresed to separate the unbound hairpins (i) from more slowly migrating DNA-protein complexes (ii and iii), and then scanned to image the labelled complexes. I1 binds to all of the hairpins we tested under these conditions, but the S hairpin more readily disrupts the I1+SΔ1Δ3–6 and I1+SΔ1–6 complexes, than the I1+S complex itself (upper panel). The distribution of the fluorescent labels in each of the these and additional EMSA experiments (including I1 interactions with the SΔ1–3 and SΔ3–6 hairpins) is also plotted to illustrate the binding curves (lower panel, average ± SD, N = 3 per data point).

All the hairpins we tested were still bound by recombinant I1 protein including SΔ (1–6) DNA, bearing a perfect hairpin (Fig 10, upper panel, lane 15). A single major shifted species was detected, although adding more protein produced greater quantities of more highly shifted protein-DNA complexes. This suggests these substrates can support multiple binding events (Fig 10, band iii). To gain insights into whether I1 exhibits differences in its *relative* affinity for the different hairpin forms, we tested how effectively these protein-DNA complexes could compete with unlabeled native S hairpin. We saw that most of the forms tested clearly exhibited a reduced affinity for I1, relative to the S hairpin itself (Fig 10, lower panel). These include the SΔ (1–6) mismatch-free hairpin (which could not be rescued into virus), the SΔ1Δ3–6 hairpin (which produced the most attenuated virus we could make) and the SΔ3–6 hairpin (which exhibited an intermediate growth phenotype). The one exception of those tested was the SΔ1–3 hairpin which behaved much like S hairpins in these competition assays, even though viruses bearing SΔ1–3 termini behaved like SΔ3–6 viruses in culture (Fig 4). It is possible that the position of the mismatch affects I1 substrate recognition since SΔ1–3 molecules, unlike the other mutant forms, still incorporate mismatches 3-to-6. A more extensive survey using quantitative binding assays, additional hairpin substrates, and I6 protein as well, is needed to sort out the binding interactions. Nevertheless, it is clear that deficiencies in I1 binding *in vitro*, relative to the S hairpin, extend to causing defects in virus growth *in vivo*.

### The sVAC-Δ1Δ3–6 hairpin virus is attenuated in vivo

**Fig 11:**
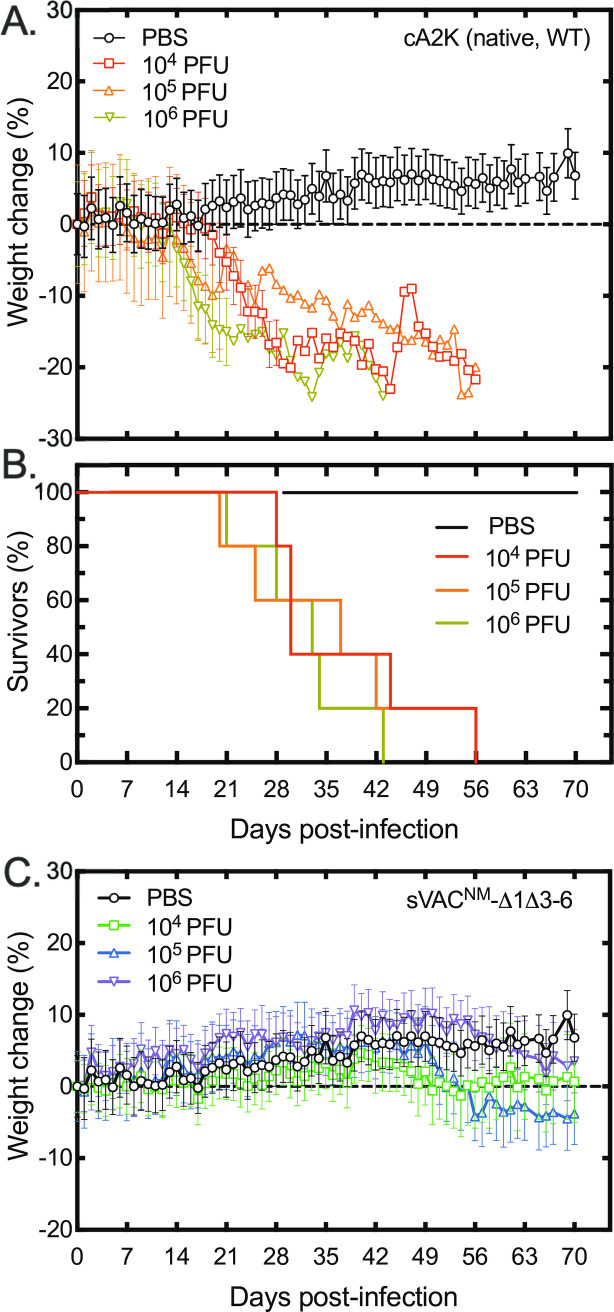
Disease in VACV-infected SCID-NCr mice. Mice were challenged by tail scarification with the indicated doses of a clone derived from a sample of Acambis 2000 virus (cA2K, panels A and B) or the sVAC^NM^-SΔ1Δ3–6 (“no marker”) virus (panel C). A PBS control experiment was also performed in parallel with the two infection trials, and has been plotted twice for reference. Virus dose had no significant effect on the differences in survival in the cA2K-treated mice with median survival ranging from 30–37 days (panel B). All of the mice infected with sVAC^NM^-SΔ1Δ3–6 virus survived over 70 days of study (panel C). The J2R locus was restored in the no marker virus to facilitate comparison with the J2R^+^ cA2K strain.

To test if these mutations affect VACV growth and virulence *in vivo*, we examined infections using both immune deficient and immune competent mice. We began by replacing the YFP-gpt markers, which attenuate the virus, with intact copies of the J2R locus. We also conducted some pilot trials to identify an appropriate mouse strain and dosing requirements. Strains cloned from Dryvax, like A2K, are avirulent in immune-competent mice but do cause disease in SCID/NCr mice [25]. The pilot trial confirmed that our cA2K clone can cause disease in SCID/NCr mice when delivered as a single dose by tail scarification, although the disease progresses slowly with half the animals reaching an endpoint defined by animal care protocols in 30–37 days (Fig 11, panels A and B). The median survival time wasn’t significantly different between the three treatment doses. By comparison, no animals reached endpoint over more than 70 days when inoculated with J2R^+^ sVAC^NM^-Δ1Δ3–6 virus although some weight loss began to be seen at later time points when compared to the PBS control (Fig 11, panel C). Again, the progression of the disease did not seem to be greatly affected by the initial dose of virus.

**Fig 12:**
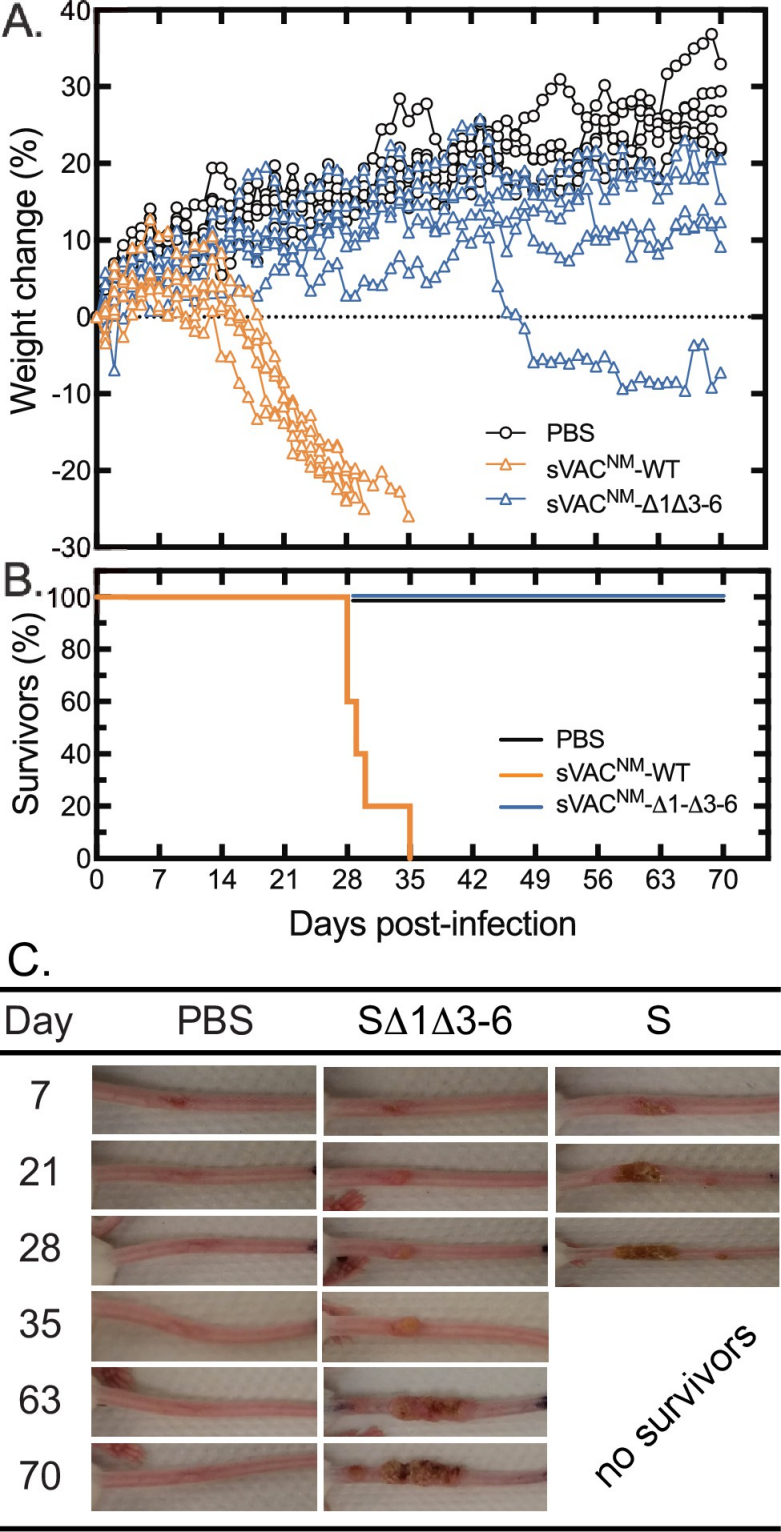
Effect of hairpin mutations on VACV virulence in immune-deficient mice. SCID-NCr mice (5 per group) were challenged with 10^5^ PFU of sVAC^NM^-wt or sVAC^NM^-Δ1Δ3–6 viruses by tail scarification. A PBS control study was also conducted in parallel. The median survival in mice infected with the sVAC^NM^-wt virus was 29 days whereas no animals were euthanized in either the PBS or sVAC^NM^-Δ1Δ3–6 groups over 70 days (panel B). The tail lesions were also photographed once per week during the experiment. Panel C shows the persistence of the lesions in representative images.

We next repeated this experiment but comparing the sVAC^NM^-wt virus to sVAC^NM^-Δ1Δ3–6 virus and using a dose of 10^5^ PFU per mouse (Fig 12). The two strains are isogenic except for the alterations in the hairpin ends. We chose 10^5^ PFU because 10^4^ PFU had previously caused only one mouse out of five in the SΔ1Δ3–6 hairpin virus group to develop a lesion at the infection site while all other animals in the cA2K (10^4^ to 10^6^ PFU) and sVAC^NM^-Δ1Δ3–6 (10^5^ and 10^6^ PFU) groups developed tail lesions. In this new experiment the median survival time for sVAC^NM^-wt strain of 29 days was not calculated to be significantly different from the 37 days previously measured with 10^5^ PFU of wildtype cA2K strain (Fig 11). This showed that neither the substitution of WR hairpin ends, nor the complete deletion of the 70 bp repeats, affected the virulence of the sVAC^NM^-wt virus in this model. Deleting the mismatched bases in the sVAC^NM^-Δ1Δ3–6 mutant reproduced the results of the previous experiment, with no mortality seen over 70 days (Fig 12B). Also as was seen in the previous experiment, several animals in this cohort of five animals began to lose weight later in the infection, one quite noticeably, with this effect beginning about 45 days into the study (Fig 12A).

SCID/NCR mice develop tail lesions at the infection site and pictures of representative lesions were taken every 7 days (Fig 12C). The lesions produced by scarification with PBS healed quickly whereas all 5/5 mice of the sVAC^NM^-wt group exhibited lesions that deteriorated quickly. In this experiment 3/5 mice from the SΔ1Δ3–6 cohort developed tail lesions whereas in the pilot trial 10^4^ PFU of SΔ1Δ3–6 virus produced lesions in 1/5 animals and 10^5^ PFU produced lesions in 5/5 animals. These data suggest that these mutant viruses not only cause fewer disease symptoms but are less infectious in the first place.

**Fig 13:**
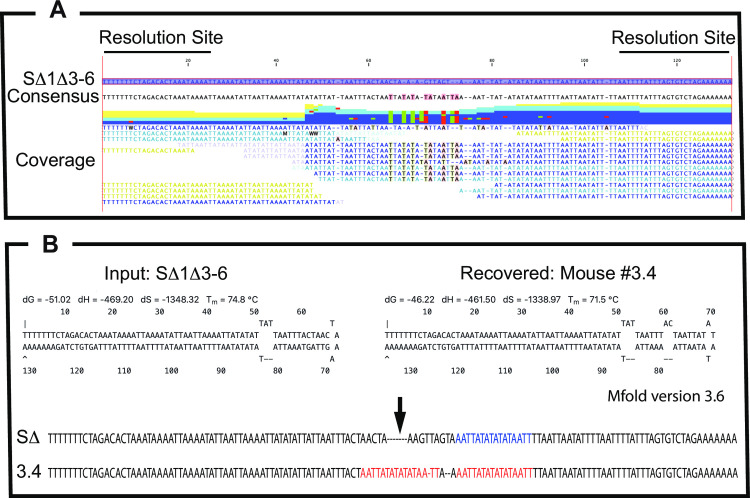
Sequence of a virus telomere recovered from a sVAC^NM^-Δ1Δ3–6 infected tail lesion. Panel A. The virus titer was expanded without a plaque purification step, purified, and sequenced. Reads derived from the hairpin region were retrieved as described previously [19] and are shown aligned with the SΔ1Δ3–6 hairpin drawn in line form. Panel B. M-fold models showing the predicted structure of the SΔ1Δ3–6 virus hairpin and the hairpin encoded by the recovered virus. The new virus seems to have duplicated a 16 nt sequence (red text) present in the original SΔ1Δ3–6 virus hairpin (blue text), and inserted it into where the original hairpin loop was found. This alteration is predicted to produce an additional mismatch and decrease the stability of the hairpin.

A feature of the disease course in both studies in SΔ1Δ3–6 infected mice is that the health of the animals began to deteriorate 4–6 weeks post infection, judging by the weight changes, (Figs 11 and 12). This led us to wonder whether the slow course of the disease might favor evolution of escape mutants. We recovered and sequenced a pool of viruses from each of the lesions that had persisted in six of the animals studied in the dose escalation trial (Fig 11). Ordinarily only a small fraction of the reads produced by Illumina methods can be mapped to the hairpin ends and the mutant hairpins proved especially difficult to sequence. However, one mouse that was inoculated with 10^5^ PFU of virus began to lose weight about 6 weeks into the infection and enough reads from what appeared to be a single population of viruses could be obtained to deduce a hairpin sequence (Fig 13A). This virus seemed to have copied a pre-existing patch of sequence into a site where the original hairpin bend would have been located, adding another mismatch and new hairpin terminus (Fig 13B). These alterations would decrease the stability of the hairpin from ΔG = -51 to -46 Kcal·mol^-1^, although the new hairpins would still be more stable that the native VACV WR hairpin (ΔG = -29 Kcal·mol^-1^). No other mutations arose outside of the telomeres over the course of the disease in these SCID/NCR mice. Unfortunately, not enough examples of other telomere mutations were detected to say, with confidence, whether this is a generalized phenomenon.

### The sVAC^NM^-Δ1Δ3–6 hairpin virus can protect mice from a lethal challenge

**Fig 14:**
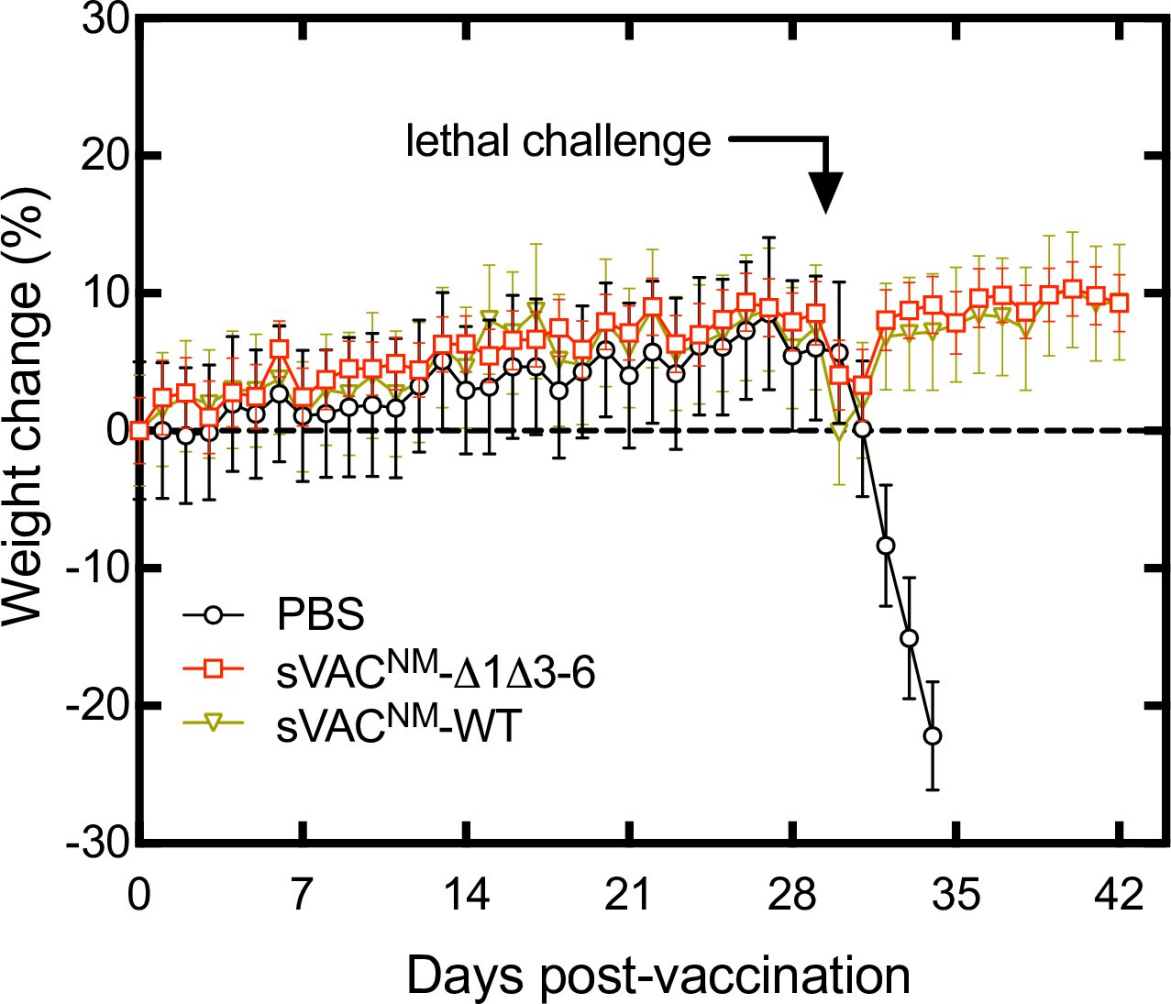
Vaccine properties of the sVAC^NM^-Δ1Δ3–6 virus. Immune competent Balb/c mice (5 per group) were infected or mock-infected with the indicated viruses by tail scarification on day zero, and then challenged with a lethal dose of intranasal VACV strain WR, 28 days later. Both synthetically-derived viruses provided an equal degree of protection again the lethal challenge.

Finally, we tested whether these mutant viruses could still vaccinate against a lethal virus challenge. We immunized Balb/c mice with 10^6^ PFU of either sVAC^NM^-wt or sVAC^NM^-Δ1Δ3–6 hairpin virus along with a PBS-treated control. All of the mice formed scabs at the scarification site which resolved by 2- or 3-weeks post-infection in the PBS/SΔ1Δ3–6 and S-treated cohorts, respectively. The mice were then challenged at day 28 post-infection with a lethal dose of VACV strain WR (Fig 14). The animals were monitored for weight loss and scored for clinical signs of illness. All of the mice that were vaccinated with either the S or SΔ1Δ3–6 hairpin viruses were fully protected against the lethal challenge, with only a transient weight loss in both groups of about 5% body weight. There were no significant differences between the two groups in any of the health parameters we measured. In contrast the PBS control cohort sickened rapidly within a couple of days of challenge and all had to be euthanized a few days later.

## Discussion

Extra-helical bases are a highly conserved and characteristic feature of all known poxvirus telomeres. In this study, we’ve shown that one cannot entirely delete these extra-helical loops without affecting virus viability. At least some of the loops are probably essential although our failure to recover viruses lacking any of the mismatches isn’t perfect proof of that contention. The difficulty in studying these hairpin ends has stemmed from an inability to modify them within the viral genome using conventional molecular techniques. Taking advantage of this new approach allowed us to study those elements *in situ*. Such an approach can open up avenues to explore the importance of other features of the viral genome that would be difficult to manipulate otherwise.

**Fig 15:**
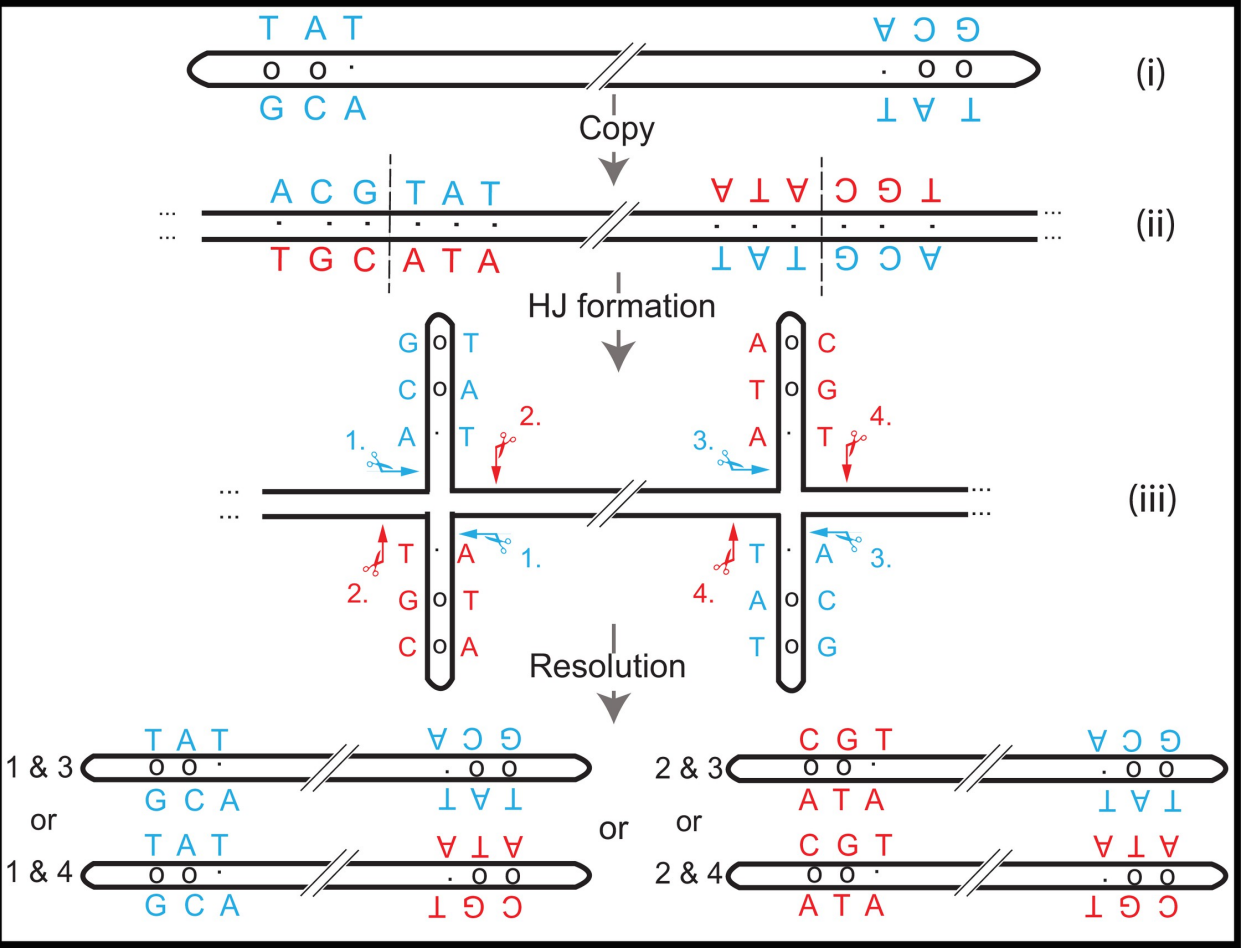
Redistribution of F- and S- poxvirus hairpin elements. Structure (i) shows a simplified view of a VACV genome bounded by two identical hairpins. These bear mismatched base pairs (blue lettering, mismatches = “o”) in an inverted repeat configuration and represents a simplified illustration of one of the starting structures assembled in this study. DNA replication is expected to generate a linear concatemer [3] composed of only Watson-Crick base pairs (ii), which can subsequently refold into a pair of Holliday structures flanking the central genome (iii). The process of branch migration reforms the mismatched base pairs and creates the virus hairpins. In VACV-infected cells these structures would be cleaved by the A22 Holliday junction resolvase [5], but depending on how these pairwise symmetrical cuts (e.g., cuts 2+2) are oriented within and between the two Holliday junctions, the reaction should produce four different arrangements of the F- and S- ends.

The assembly reactions also offered an interesting insight into the arrangement of F and S hairpin ends in VACV. The first steps in the reactivation reactions used DNA substrates encoding the left and right terminal inverted repeats, but with both bearing only one of the two ends. Yet the pool of viruses that were recovered later, once again encoded both F and S hairpins. The result may seem counterintuitive but it is not surprising given how Holliday junctions are resolved [26] (Fig 15). It suggests that VACV stocks actually comprise four sub-populations of genomes that collectively encode all four possible arrangements of the two ends.

Our results strongly support the hypothesis that VACV telomere function is more dependent upon structure than on sequence as an SFV hairpin, with a different linear sequence, can fully substitute for a VACV hairpin. Hairpin function is fairly tolerant of sequence changes as one can delete several mismatches from this ~70 bp region before it begins to affect the recovery or growth of the virus. The SΔ1Δ3–6 virus encoded a single loop plus the hairpin end and appears to mark the “end of the line” insofar as neither a perfect hairpin, nor two other single loop forms permitted recovery of viable viruses (Fig 3). The SΔ1Δ3–6 virus grew to lower titers, but exhibited no defects in DNA replication or concatemer resolution. In that regard it phenocopies the previously reported effects of mutations in one of the VACV telomere binding proteins. In particular, cells infected with the SΔ1Δ3–6 virus produce an abundance of IV-like particles (Fig 7) and a similar effect was seen when I1 expression is blocked [15]. This is consistent with our observation that the SΔ1Δ3–6 hairpin exhibits a reduced affinity for recombinant I1 protein relative to the native S-hairpin (Fig 10). However, one difference between the morphological effects of hairpin and I1L mutations is that SΔ1Δ3–6 viruses are still infectious whereas virus made in the absence of I1 are not. The simplest explanation is that the SΔ1Δ3–6 mutation is incompletely penetrant. Indeed, one can detect a small proportion (2–3%) of MV-like particles in the SΔ1Δ3–6 stocks, which suffice to explain the residual infectivity.

Our EM, flow virometry, and qPCR experiments all showed that the SΔ1Δ3–6 viruses contained DNA which is different from the effect seen when one disrupts I6 function. Those experiments with I6 produced virus particles containing electron-dense cores but lacking DNA [14]. The differences in phenotypes suggest that I6 operates in a manner epistatic to I1 and that the SΔ1Δ3–6 mutation interferes with latter-stage events dependent on I1. Our work is still fully consistent with the hypothesis that the hairpin mismatches signal to systems that collectively mediate DNA loading, virion assembly, core protein processing (Fig 9), and virus maturation [12,14,15]. However, since the approach described here can be used to test hypotheses arising from biochemical investigations into how I1 and I6 interact with hairpin variants, our new studies point to ways of further connecting and dissecting the chain of protein binding and signaling events mediated by VACV telomere binding proteins.

Unsurprisingly, the growth defect exhibited by the sVAC-Δ1Δ3–6 virus caused its attenuation in mice. Nonetheless, the virus was still protective as a vaccine against a lethal VACV challenge in an immunocompetent mouse model. Whether such alterations offer a practical way to attenuate a vaccine vector is uncertain as at least one of the sVAC^NM^-Δ1Δ3–6 viruses acquired new hairpin mutations over the course of ~70 days in immune compromised mice. No other mutations arose anywhere else in the synthetically assembled genome. The fact that mutant poxviruses are under genetic pressure to restore these extrahelical and mismatched sequence elements *in vivo*, provides formal evidence that the structures first described nearly 40 years ago [1] do serve a function of great biological importance.

## Materials and methods

### Ethics statement

Experimental studies relating to the construction and propagation of synthetic VACV strains were reviewed by the Office of Environmental Health & Safety with regards biosafety, biosecurity, and dual-use considerations. The University’s approval was assigned under RES0040457. All of the animal studies were conducted under the auspices of protocol AUP00000506. The protocol was reviewed and approved by the University’s Research Ethics Office, Animal Care and Use Committee, in accordance with the guidelines and policies of the Canadian Council on Animal Care (https://www.ccac.ca).

### Cells, viruses, and other reagents

VACV (strain WR), Shope fibroma virus, African green monkey cells (BSC-40), and Buffalo green monkey kidney cells (BGMK) were originally obtained from the American Type Culture Collection. VACV (strain Acambis 2000) was a gift from Dr. M. Buller (St. Louis, MO). A clone was plaque purified, sequenced, and our laboratory’s isolate (cA2K) has been assigned Gen Bank accession number MN974380. The viruses and cells were cultured at 37°C in 5% CO_2_ in minimal essential media (MEM) supplemented with antibiotics, L-glutamine, nonessential amino acids, sodium pyruvate, and 5% fetal bovine serum (GIBCO) or fetal bovine serum plus Fetal Gro (RMBIO). All cell lines tested negative for mycoplasma. Western blots used a rabbit polyclonal antibody directed against VACV A3 (p4b/4b) protein. It was a kind gift from Dr. B. Moss, NIH. An Odyssey imager and secondary antibodies (LI-COR) were used to detect the proteins.

### sVAC-wt virus design and reactivation

Synthetic copies of cA2K (sVAC-wt) were assembled as described previously [19] using 9 large fragments of DNA (Fig 1). These were a gift provided by Tonix Pharmaceuticals, New York. The left and right end fragments were separately ligated to synthetic oligonucleotides encoding WR hairpins and CRS (the CRS is identical in both viruses), plus a 64 bp linker also derived from VACV strain WR. The design of the linkers and a sequential pattern of restriction digests were used to direct the order and sites of addition of the different elements. More specifically the hairpin oligonucleotides encoded a 5’-ACA-3’ single-stranded extension compatible with a 5’-TGT-3’ extension on one end of the 64 bp linker. The other end of the linker encoded a 5’-TGG-3’ extension compatible with the sticky end left by cutting the plasmids encoding the left or right TIR fragments with *Sap*I. The resulting viral ITRs encode eight 54 bp and two 125 bp repeats, but none of the 70 bp repeats. Most of the *Bsa*I sites were silently mutated to facilitate gene synthesis. Table 2 lists the different viruses, the sequences and hairpin fragments used to produce them (as referenced in Genbank files), and other features of each virus. Fragment 3 (Fig 1) encoded a YFP-gpt selection marker in the thymidine kinase (J2R) locus [22]. This was deleted from the viruses that were used in mice by transfecting a DNA fragment encoding the J2R gene plus flanking sequences into virus-infected cells and selecting non-fluorescent plaques. These J2R^+^ recombinants are identified as “no marker” viruses (e.g. sVAC^NM^) in this communication. Fluorescent forms of the J2R^+^ strains were subsequently reconstructed by replacing the native A5L gene with one encoding YFP fused to the N-terminus of the A5 open reading frame [23].

### Virus stocks

All VACV stocks were grown and titered on BSC-40 cells. The experiments used viruses prepared by releasing the virus from the cells using a Dounce homogenizer in 10mM Tris·HCl pH 9 plus 2 mM MgCl_2_ buffer, and treating the suspension for 30 min at 37°C with 50 U/mL benzonase (Millipore Sigma). The treated preparations were then purified and concentrated by centrifugation at 26,000 x g for 90 mins onto a 36% sucrose cushion. After resuspending the virus pellet is 10 mM Tris·HCl pH 8, it was sonicated in ice water using a cup-horn sonicator (Qsonica Sonicators) for 1.5 min in 3 x 30 sec pulses. The stocks were filtered through a 70 μm filter, aliquoted, and stored frozen at -80°C.

### Hairpin characterization

Hairpin-forming oligonucleotides (Table 1) were purchased from GenScript (Piscataway, NJ), dissolved in water, heated at 95°C for 5 min, and quick cooled on ice for 10 min to facilitate hairpin formation. The Mfold web server with default settings [27] [http://www.unafold.org] was used to model possible DNA folding patterns. To validate the predicted secondary structures, reactions containing 10 U of mung bean nuclease (Promega), 500 ng of each DNA, 50 mM potassium acetate, 20 mM Tris acetate, 10 mM magnesium acetate, and 100 μg·mL^-1^ BSA (pH 7.9) was incubated for 1 hour at 37°C then fractionated on a 4% agarose gel.

### Virus growth and DNA replication

Multistep growth curves were used to measure virus yields. BSC-40 cells were infected with virus at MOI = 0.01 PFU/cell, scraped into the media at the indicated times over the next 3 days, freeze/thawed, diluted, and the virus titered on BSC-40 cells. Single-step growth curves were used to evaluate genome replication. BSC-40 cells were infected with virus at MOI = 3 PFU/cell and harvested at different time points over the next 24 hr. Whole-cell DNA was isolated and quantitated using a Nanodrop spectrophotometer. A qPCR protocol [28] was used to determine the quantities of virus DNA. Each assay used 100ng of DNA and two E9L primers (5’ CTCTGCTCCATTTAGTACCGATTC 3’ and 5’ TACTCATACGCTTCGGCTAAGA 3’) plus a double-quenched probe purchased from Integrated DNA Technologies (5’ /**56-FAM**/AGATCATTC**/ZEN/**TACGTCCTATGGATGTGCAAC/ **3IABkFQ**/ 3’). A gene block consisting of the following sequence was used to calibrate the assay: 5’ GGATTGGCAAACCGTAACATACCGTTAGATAACTCTGCTCCATTTAGTACCGATTCTAGATACAAGATCATTCTACGTCCTATGGATGTGCAACTCTTAGCCGAAGCGTATGAGTATAGAGCACTATTTCTAAATCCCATCAGACCATAT 3’.

### Concatemer resolution analysis

DNA was extracted from virus-infected cells at different times after infection, digested with *Alw*44I (Thermofisher), size fractionated by electrophoresis through a 0.8% agarose gel, and imaged using SYBR Gold stain. The DNA was transferred to a Biodyne B Nylon membrane, crosslinked with UV-light, hybridized, washed, and detected using a GE ImageQuant LAS-4000 imager. The biotin-labelled probes were prepared using PCR plus 5’ AGACACACGCTTTGAGTTTTG 3’ and 5’ GATTCTTCCTCCAAACAGTTAACG 3’ primers, as directed by a North2South chemiluminescent detection kit (Thermofisher). The bands detected in the digital gel images were quantitated using Fiji v.2.2.0 (https://imagej.nih.gov/ij) and the signal intensities plotted using Microsoft Excel (v16.57) and Graph Pad Prism (v9.3.1).

### Flow virometry

The sVAC^NM^-Δ1Δ3–6 virus was purified to a starting titer of 4x10^8^ PFU/ml and the sVAC^NM^-wt virus produced yields about ten-fold higher. The virus stocks were quick-thawed, sonicated, and diluted in phosphate-buffered saline (GIBCO) that had been twice filtered through a 0.1 μm filter. After preparing serial ten-fold dilutions of each virus, an equal quantity of 0.1 μm filtered 4% paraformaldehyde was added and fixed for 30 min on ice. SYBR Gold dye was added (where indicated) to the samples, to a final concentration of 2.5×, and then held on ice for at least 30 min in the dark awaiting analysis. The virus preparations were each diluted 10-fold in filtered PBS and applied to a Cytoflex Flow Cytometer (Beckman Coulter).

### Hairpin binding assays

A codon-optimized copy of the I1L gene was purchased as a clone in a pET151/D-TOPO expression vector (Thermofisher) and used to transform a *recA* derivative of *E*. *coli* strain BL21 DE3 pLysS. Recombinant I1 protein was prepared using the auto-induction method [29] and cells grown at 37°. After overnight incubation, the cells were recovered by centrifugation, resuspended in loading buffer (0.5 M NaCl, 20 mM sodium phosphate, 20 mM imidazole, pH 7.4), broken with a French press, recentrifuged, and the supernatant applied to a 1 mL GE His-trap column connected to an AKTA Pure HPLC. The column was washed and eluted with a 0.04-to-0.5 M imidazole gradient in 0.5 M NaCl, 20 mM sodium phosphate. The protein was concentrated using Amicon U-15 centrifugal filters, quantified, and stored frozen in small aliquots after dilution into a buffer containing 0.1 mM EDTA, 50 mM Tris·HCl pH 7.5, 100 μg/mL BSA, 10 mM 2-mercaptoethanol, 20 mM NaCl, and 5% w/v glycerol.

The mismatch-containing hairpin oligonucleotides (Fig 3) were 3’-end labelled in 50 μL reactions containing 5 μg oligonucleotide, 100 μM Alexa Fluor 647-aha-dUTP (ThermoFisher), 200 μM each of dATP, dCTP, and dGTP, 200 U Superscript II reverse transcriptase, and 1× first-strand buffer (Invitrogen). After 30 min at 37°, EDTA was added to 4 mM, the samples were heated at 70° for 10 min, and purified by centrifugation through G50 spin columns (GE) in 10 mM Tris·HCl, 1 mM EDTA pH 8.

The electrophoretic mobility shift assays used conditions and protocols described previously [12] except the voltages and volumes were scaled down for BioRad mini-gels and we used a 29:1 mix of acrylamide to bisacrylamide (BioRad). Each 10 μL binding reaction contained ~100 ng of labelled DNA, 10 mM Tris·HCl pH 7.5, 100 mM KCl, 0.2 mM EDTA, 0.5 mM dithiothreitol, 10% w/v glycerol, 2 μg BSA, 700 ng poly·d(IC), unlabelled hairpin DNA (as indicated), and 100 ng purified I1. After adding the I1 protein, the reactions were incubated for 30 min at 37°, loaded onto 6% acrylamide gels, electrophoresed in ice-chilled tanks for 2 hr at 50V, and imaged using the Cy5 settings on a Typhoon imager. Gel and tank buffers contained 50 mM Tris·HCl, 380 mM glycine and 2 mM EDTA, pH 8.3.

### Electron microscopy

The cells were grown to 80% confluence on ACLAR film (Ted Pella), infected with sVAC^A5-YFP^-wt or sVAC^A5-YFP^-Δ1Δ3–6 at MOI = 3 for 24hr, washed, and fixed overnight with 2% paraformaldehyde and 2.5% glutaraldehyde in 0.1 M sodium-cacodylate buffer containing 2mM CaCl_2_ (pH 7.4). The cells were washed with cacodylate buffer and post-fixed for 30 minutes in 1% K_4_FeCN_6_ and 1% OsO_4_ in cacodylate buffer. The cells were washed into 0.1M sodium acetate (pH 5.2) and treated with 2% uranyl acetate in acetate buffer for 15 minutes. The cells were washed again in acetate buffer and water and dehydrated in a graded series of 30–100% ethanol. The cells were infiltrated with Spurr’s resin (EMS) in ethanol and polymerized in 100% resin at 65° for 48 hr. The blocks were sectioned to 70 nm with an ultramicrotome (Leica, EM UC6) and post-stained with uranyl acetate and lead citrate. Images were captured with a JEOL F21000 electron microscope operating at 200kV acceleration voltage and equipped with a Gatan Orius camera.

For analysis of virus particles, each virus preparation was centrifuged through a 36% sucrose cushion at 26,000×g for 90 min and the pellet fixed with 2.5% glutaraldehyde in cacodylate buffer for 20 min at 37° then 40 min at 20°. The pellet was gently washed three times with cacodylate buffer and resuspended with one volume of 7% low gelling agarose, allowed to set, and then processed through secondary fixation with osmium tetroxide, dehydration and embedding as described above.

All microscopy was performed at the University of Alberta Cell Imaging facility. Digital images were labeled and analyzed using Fiji v.2.2.0 (https://imagej.nih.gov/ij) and assembled using Adobe Photoshop (2022) and Illustrator (2022). Fiji was used to determine the total particle counts in each field and then two independent observers, using two different pairs of images, estimated the number of MV particles in sVAC^A5-YFP^-Δ1Δ3–6 specimens and IV-like particles in sVAC^A5-YFP^-wt specimens.

### Animal care

Female NCI SCID-NCr mice were purchased from Charles River Laboratories at 6–8 weeks of age. These mice exhibit defective B and T lymphocytes, but normal NK cells, macrophage, and granulocytes. The mice were housed in BSL-2 containment and in conditions as detailed in Umer *et al*. [30]. At day 0 the mice were anesthetized and 5 μl of PBS or viral inoculum was applied with a 10 μL pipette tip to a 1 cm scarification zone near the base of the tail. The inoculum was left to dry for 5 min and then the mice were placed in a recovery chamber to monitor for signs of distress. The mice were subsequently monitored daily for weight, physical appearance, mobility, responsiveness, and pox lesions. The tail scars were imaged weekly. The mice were euthanized if they reached a total clinical score of 7, or a score of 3 on any of the individual criteria, or if they lost ≥20% of their initial body weight. Any lesions found on the tail at the injection site were dissected at euthanasia and stored at -80°. The samples were subsequently thawed, placed in a pre-wetted 70μm cell strainer, and mashed through the strainer using a syringe plunger followed by two washes with 0.5 ml each of Hank’s Balanced Salt Solution (GIBCO). Any cell-associated viruses were released by freeze-thaw and plated on BSC-40 cells.

Immunocompetent Balb/c mice were purchased from Charles River Laboratories at 6–8 weeks of age and immunized on Day 0 as described above. On Day 28 the mice were anesthetized and challenged with 10^6^ PFU of intranasal VACV strain WR. The mice were subsequently weighed and monitored daily for signs of disease and distress as described above.

### Data analysis

SnapGene (v. 5.1) was used for all of the DNA design work as well as the sequence alignments, and the genome assemblies were compiled and prepared using CLC Genomics Work Bench. Illumina-based DNA sequencing and assembly was performed as described [19]. The flow virometry data were analyzed using Flow Jo (v10.6.0) and Graph Pad Prism (v. 8.4.2) was used to analyze and graph the data. Log-rank and Gehan-Breslow-Wilcoxon tests were used to compare differences between the survival curves. All error bars show standard deviations.
